# The probabilistic and dynamic nature of perception in human generalization behavior

**DOI:** 10.1016/j.isci.2025.112228

**Published:** 2025-03-17

**Authors:** Kenny Yu, Wolf Vanpaemel, Francis Tuerlinckx, Jonas Zaman

**Affiliations:** 1 Quantitative Psychology and Individual Differences, KU Leuven, 3000 Leuven, Belgium; 2 REVAL Rehabilitation Research, Faculty of Rehabilitation Sciences, UHasselt, 3590 Diepenbeek, Belgium; 3 Centre for Learning and Experimental Psychopathology, KU Leuven, 3000 Leuven, Belgium; 4 Center for Translational Neuro- and Behavioral Sciences, University of Duisburg-Essen, 47057 Duisburg, Germany

**Keywords:** Social sciences, Psychology

## Abstract

Generalization theories traditionally overlook how our mental representations dynamically change in the process of transferring learned knowledge to new contexts. We integrated perceptual and generalization theories into a computational model using data from 80 participants who underwent Pavlovian fear conditioning experiments. The model analyzed continuous measures of perception and fear generalization to understand their relationship. Our findings revealed large individual variations in perceptual processes that directly influence generalization patterns. By examining how perceptual and generalization mechanisms work together, we uncovered their combined role in producing generalization behavior. This research illuminates the probabilistic perceptual foundations underlying individual differences in generalization, emphasizing the crucial integration between perceptual and generalization processes. Understanding this relationship enhances our knowledge of generalization behavior and has potential implications for various cognitive domains including categorization, motor learning, language processing, and face recognition—all of which rely on generalization as a fundamental cognitive process.

## Highlights

• Human perception varies between individuals and within individuals over time
• Probabilistic perception modulates how past fear experiences generalize to new situations
• Cognitive–perceptual interplay drives fear generalization
• Computational modeling connects generalization and perceptual theories

## Graphical abstract

**Figure:**
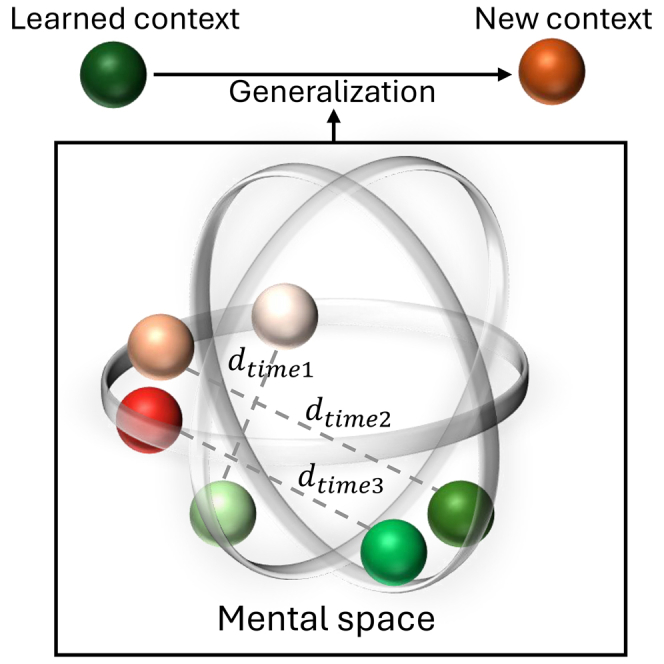

## Introduction

Humans possess a remarkable ability to extrapolate past learning to new situations, a cognitive process known as generalization.^1^ This adaptive mechanism allows individuals to transfer knowledge efficiently, avoiding the need to relearn in similar contexts. Generalization relies heavily on the concept of similarity: the more alike two situations are, the more readily knowledge is transferred between them.^2^ However, humans do not interact with a physical reality directly. Instead, we perceive the world through mental representations shaped by our sensory systems. These mental representations form the foundation for assessing similarity and guiding generalization. Crucially, these representations vary both between individuals and within the same individual across different instances, leading to idiosyncratic patterns in how similarity is assessed and generalization occurs.^3^

**Figure 1:**
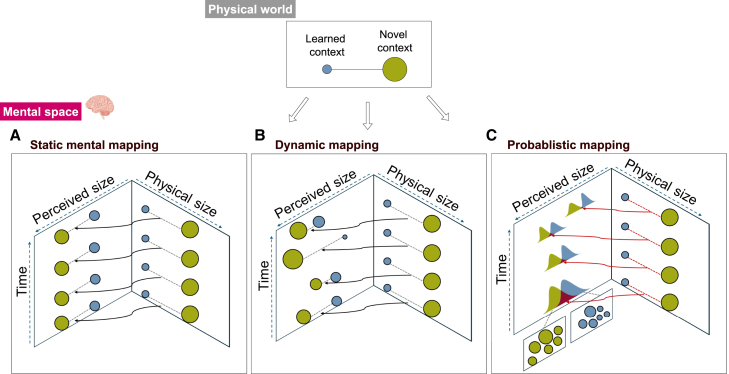
From physical world to mental space (A) The mental distance between two contexts is determined by a one-to-one mapping between physical to perceptual quantity. (B) The mental distance has a temporal dynamic that shifts over time. (C) The mental distance has a dynamic and stochastic nature.

However, a prevalent trend in generalization research is the tendency to conflate the mental with the physical, often treating these dimensions as synonymous entities.^1^^,^^4^ While some researchers have acknowledged the inherent noise in the perceptual process^5^ or recognized that the mental representation may deviate from the physical reality (Figure 1A),^2^ they often overlooked three critical aspects of perception: the variability across individuals, the changes within individuals over time, and how learning processes themselves can modify perception.^6^

As organisms, we perceive the physical world indirectly through sensory inputs across various modalities.^7^ These inputs undergo complex mental processing to form perceptual judgments.^8^^,^^9^ The predictive coding framework^10^^,^^11^ within Bayesian brain theory^12^^,^^13^^,^^14^ provides a compelling explanation for this process: the brain functions as an inference system that generates predictions based on environmental expectations. When encountering new information, perception involves integrating these new experiences with past predictions. The degree of updating depends on the relative uncertainty – greater updating occurs when sensory evidence is more certain or prior knowledge is uncertain. While debate continues about whether human perception optimally follows Bayes’ rule,^15^^,^^16^ substantial evidence supports that past experiences shape current perception.^17^^,^^18^^,^^19^^,^^20^ Consequently, mental representations of stimuli exist not as fixed points but as evolving probability distributions, with individual differences in how sensory input and expectations combine.^21^

In generalization research, probability distributions have been used to explain how overlapping features of stimuli influence generalization.^22^^,^^23^^,^^24^ For instance, when two stimuli share similar features, their representational distributions overlap, leading to a higher likelihood of generalization. However, all traditional theories assume the mental representation of a stimulus has a fixed shape and spread that remains constant over time and is identical across different people (Figure 1A). This traditional approach faces a fundamental limitation: by constructing distributions solely from generalization behavior, it becomes impossible to disentangle the distinct cognitive components that shape generalization. This creates a critical theoretical inference problem, as researchers cannot determine whether observed generalization patterns stem from perceptual or memory processing differences. Given that overgeneralization has been consistently linked to anxiety-related disorders,^25^^,^^26^^,^^27^ this theoretical limitation has profound clinical implications. Without the ability to isolate specific cognitive components driving generalization, interventions designed to modify generalization patterns may fail if they target the wrong cognitive component. For instance, a treatment approach focused on modifying response tendencies might prove ineffective if the patient’s anxiety-related overgeneralization primarily stems from altered perceptual processing^28^. Previous research provided evidence for this perceptual account by showing that anxiety patients exhibited overgeneralization partly due to altered sensory representations.^29^

Recent empirical studies have begun addressing these limitations by revealing two key mechanisms underlying generalization behavior: perceptual processing^30^^,^^31^^,^^32^^,^^33^^,^^34^ and memory operations.^35^^,^^36^^,^^37^^,^^38^ These findings demonstrate the inadequacy of traditional generalization theories by showing how individuals may perceive the same physical features differently, and how their memories of these perceptions can shift over time (Figures 1B and 1C). For example, Zaman et al. (2023)^36^ found large individual differences in how people perceive colors and identify previously learned stimuli, with these idiosyncratic patterns strongly predicting their generalization behavior. This dynamic variability suggests that to truly understand generalization and develop effective treatments, we need methodologies that can separately measure and model the distinct contributions of perceptual processing and memory mechanisms, rather than conflating them within a single distribution derived from behavioral outcomes alone.

**Figure 2:**
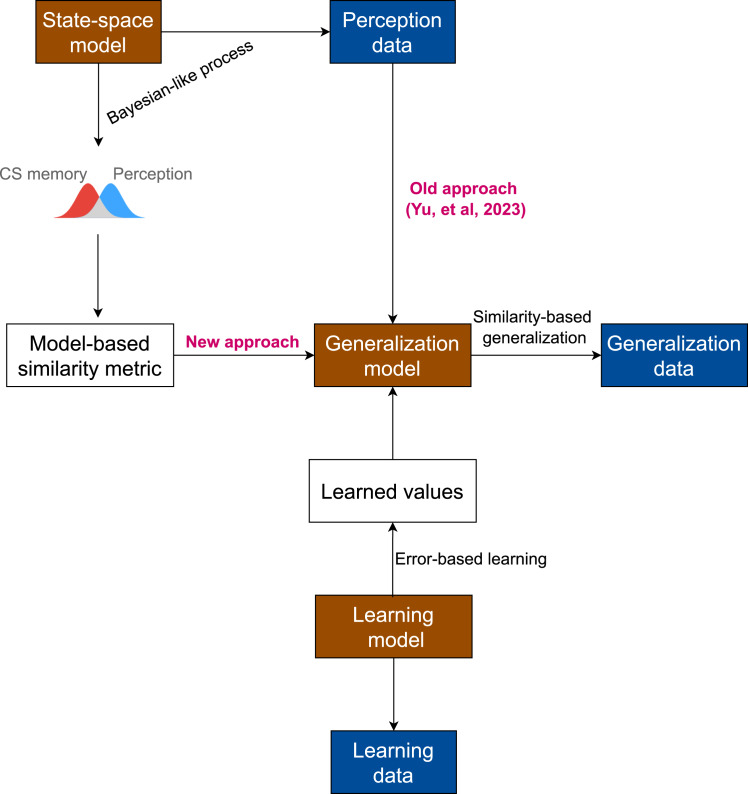
Model framework overview The computational framework integrates a state-space model that processes perceptual data in a Bayesian-like manner, generating perceptual and CS memory distributions. These distributions inform a model-based similarity metric, which feeds into the generalization model alongside learned values from the learning model. This new approach contrasts with the previous point-based perceptual representation for similarity-based generalization.

Recently, we introduced a computational model^3^ that made a first attempt to explicitly include perception into the generative process underlying fear generalization (Figure 2). It departs, like most generalization theories, from an error-driven learning process^39^ that captures how organisms adapt their behavior based on experience. When an outcome differs from what was expected, learners update their predictions to minimize future errors – larger surprises lead to bigger adjustments in their expectations. Generalization of these learned expectations follows an exponential decay principle:^2^ the more different two situations are, the less likely learning will transfer between them. This decay occurs within a mental space where stimuli are represented based on their perceptual features. In traditional theories, this mental space is assumed to be static and uniform – stimuli occupy fixed positions that remain constant over time and are identical across individuals. Our model departs from this assumption by using trial-by-trial perceptual data to track how individuals’ mental representations of stimuli change over time.^3^ This allows stimulus positions in mental space to shift dynamically and differ between individuals. Consequently, the similarity between any two stimuli, which is derived from their distances in this space, becomes dynamic rather than fixed. This enables the model to account for variations in generalization patterns based on how individuals uniquely represent stimuli in their mental space. Crucially, unlike previous approaches that inferred these representations from generalization behavior itself, our model derives them independently from trial-by-trial perceptual data, avoiding the circularity inherent in earlier theories.

While this model successfully accounts for individual differences in generalization behavior,^3^ it nevertheless implements a reductionist approach to perception that warrants further refinement. The model’s representation of perception as discrete points in mental space fails to capture two critical aspects of perceptual processing. First, it overlooks the inherent uncertainty in sensory processing, where each percept exists not as a singular value but as a probability distribution reflecting the brain’s confidence in its interpretations.^13^^,^^14^^,^^20^ Second, it does not account for the cumulative nature of perceptual experience, whereby current perceptions are actively shaped by the integration of past perceptual encounters with incoming sensory information.^17^^,^^18^^,^^19^^,^^20^ This simplified conceptualization of perception, while enabling initial insights into individual differences in generalization, ultimately constrains our ability to fully elucidate the fundamental perceptual mechanisms that underlie generalization behavior and their dynamic interaction with learning processes.

In this study, we introduce a fundamental shift in understanding how perception shapes generalization by resolving a critical limitation in existing theories. While early theories assumed direct access to physical stimulus properties (Figure 1A), later approaches acknowledged uncertainty through probability distributions but could only infer these distributions from generalization behavior itself - creating a circular explanation. Our previous work^3^ began addressing this circularity by incorporating direct perceptual measurements, but treated each perception as a precise point rather than an uncertain estimate (Figure 1B). We now present a framework that captures two fundamental aspects of human perception: its inherent uncertainty and its continuous evolution through experience (Figure 1C). By modeling perceptual representations as dynamic probability distributions derived from trial-by-trial perceptual judgments (Figure 2), we allow similarity to emerge naturally from the overlap between uncertain perceptual representations rather than inferring it from behavior or physical properties. To ensure rigorous comparison between probabilistic and traditional approaches, we implement a two-step analysis: first modeling perceptual uncertainty using only perceptual data, then using these pre-computed similarities in our generalization model. This separation prevents our probabilistic assumptions from biasing model comparison while still capturing how both immediate perception and accumulated experience shape generalization behavior.

To investigate these new assumptions, we re-analyzed data from two published fear conditioning experiments (total N=80)^3^ where participants rated the size of circular stimuli and their expectancy of receiving an electrocutaneous stimulus. In Experiment 1, participants underwent simple conditioning with one reinforced circle (CS+) paired with shock. In Experiment 2, differential conditioning was used with both a reinforced (CS+) and non-reinforced (CS−) circle. During generalization testing, participants rated their shock expectancy for new circles of varying sizes. Both experiments provided continuous measures of perception (size ratings) and fear learning (shock expectancy) throughout conditioning and generalization phases.

## Results

We contrasted two computational approaches that differed on their operationalization of the mental representation of stimuli. The first approach presumed stimuli as points with their coordinates directly derived from the perceptual judgment data (Figure 1B), replicating the methodology established in the previous study.^3^ The second approach conceptualized stimuli as probabilistic perceptual distributions that are the result of a Bayesian perception process that integrates past perceptual experiences and sensory input (Figure 1C).

In the following sections, we will first explore the inter- and intra-individual patterns observed in perceptual judgment data using the Bayesian perceptual model. Subsequently, we will explore how these perceptual patterns can be effectively used to predict generalization patterns.

### Perception

Our investigation into perception centered on two key aspects: (1) understanding how individuals uniquely transform physical stimuli into subjective perceptual experiences through personalized sensory mappings (perceptual likelihood), and (2) examining the role of individual differences in accumulated perceptual history (perceptual prior) in shaping subsequent judgments. To model human perception, we employed a state-space framework using a Kalman filter—a mathematical model that captures how individuals integrate prior experiences with incoming sensory information. In essence, this approach reflects how the brain not only processes raw sensory input but dynamically combines it with past perceptions to form coherent judgments. The Kalman filter quantitatively accounts for the relative reliability of new sensory evidence and pre-existing perceptual beliefs, providing a principled mechanism to model this interplay (for detailed explanations, refer to section perceptual model).

Before delving into modeling the data collected from actual experiments, we initiated a simulation study where we generated perceptual responses for 300 synthetic participants. This simulation allowed us to explore parameter recovery and determine if the model could accurately identify parameter values. Encouragingly, the simulation results indicated successful recovery of all critical parameters within the models (Method S1: Parameter Recovery and Figure S1).

**Figure 3:**
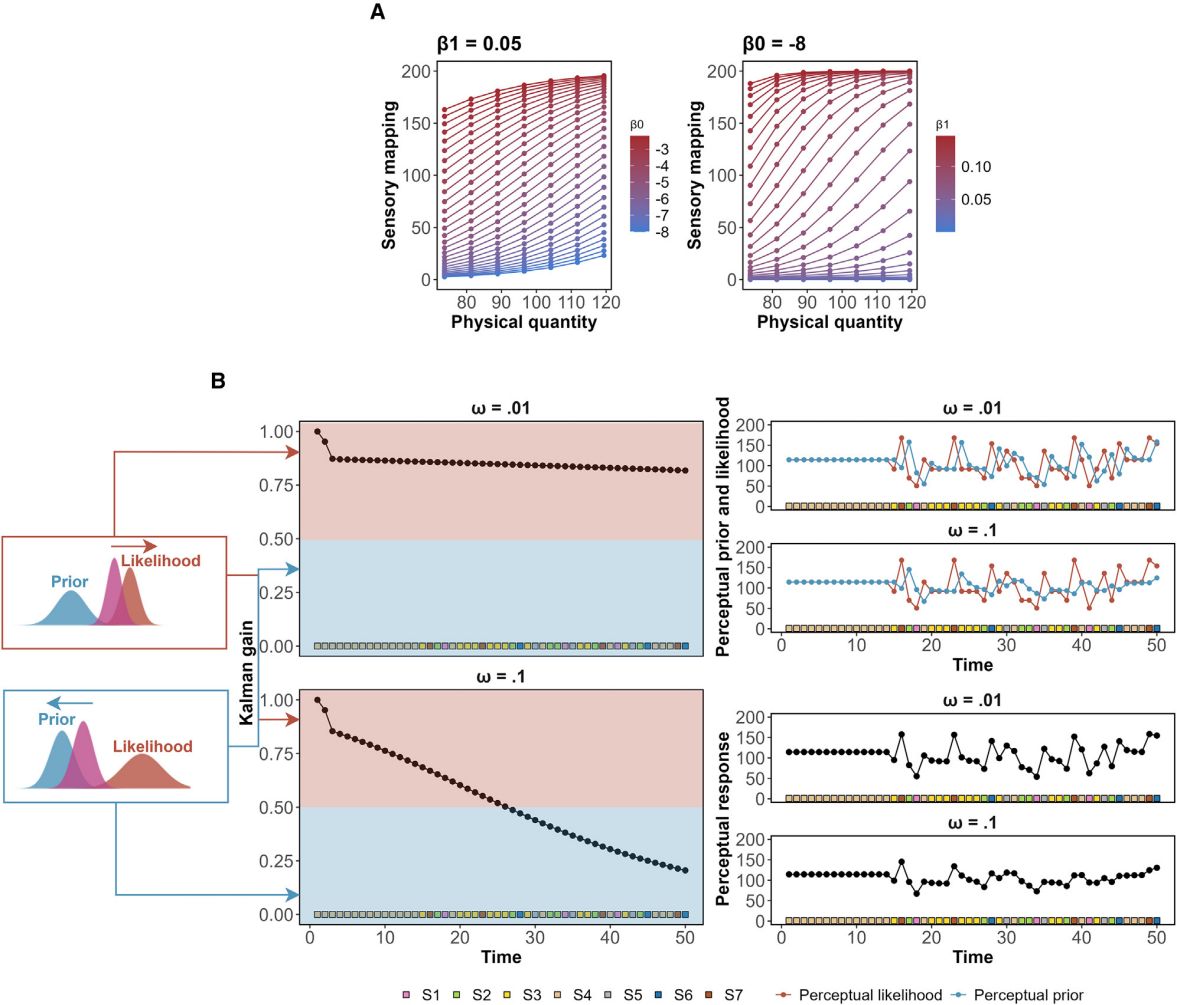
Patterns in perceptual model (A) The combination of the two scaling parameters $\beta_{0,i}$ and $\beta_{1,i}$ in the sigmoid function can generate different patterns of perceptual mappings. (B) The determination of the perceptual mean at each time point involves a weighted combination of the mean values from the perceptual prior and the mean values from the perceptual likelihood (sensory mapping). The perception at a given time point is more influenced by the perceptual prior when the Kalman gain remains below 0.5, while it tends to be more heavily shaped by the perceptual likelihood as the Kalman gain surpasses 0.5. The effectiveness of this transition is intricately tied to the process noise forgetting rate denoted as *ω*, which essentially determines the pace at which the Kalman gain decreases with increasing perceptual instances.

The architecture of the model centers on several key parameters that capture individual differences and temporal dynamics in perception. At the core are three participant-specific parameters: the scaling parameters ($\beta_{0,i}$ and $\beta_{1,i}$) define how physical stimulus properties are transformed into mental representations through a sigmoid function, with $\beta_{0,i}$ determining the baseline perceptual magnitude and $\beta_{1,i}$ controlling the steepness of the transformation. This initial transformation includes inherent sensory mapping uncertainty ($\sigma_{S,i}$), reflecting how consistently an individual perceives the same physical stimulus. The resulting perceptual distributions are characterized by their mean ($\mu_{\psi ,ij}$) and uncertainty ($\sigma_{\psi ,ij}$), which evolve over time. The rate of this evolution is controlled by the process noise decay parameter ($\omega_{i}$), which determines how quickly perceptual uncertainty diminishes with repeated exposure to stimuli. A higher value of $\omega_{i}$ indicates a rapid decrease in uncertainty, leading to more precise perception over time, while a lower value maintains higher perceptual uncertainty throughout the experimental session. Together, these parameters provide a comprehensive account of both stable individual differences in perception and how perceptual precision dynamically evolves through experience (see Figure 3).

#### Sensory mapping

**Figure 4:**
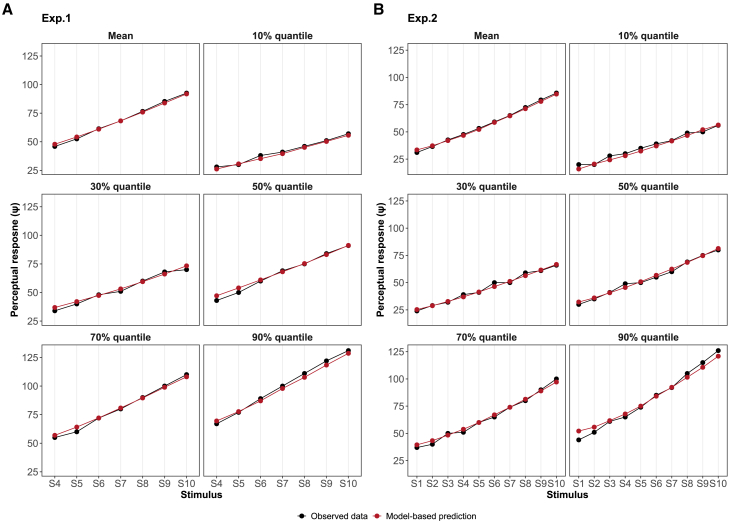
Posterior predictive checks of model-based perception Comparisons are drawn between posterior predictive samples and actual perceptual response data in both simple conditioning (A) and differential conditioning (B) experiments. The black curve represents the mean as well as the quantiles at 10%, 30%, 50%, 70%, and 90% across diverse stimuli for observed perceptual responses. In comparison, the orange curve illustrates the mean along with corresponding quantiles at 10%, 30%, 50%, 70%, and 90% for 5000 replicated data across various stimuli.

In Figure 4, we compared the perceptual responses observed in the experimental data with the replicated data generated from the posterior distribution of the parameter space. This comparison aimed to gauge the extent to which the perceptual model could accurately mimic the observed perceptual responses. As shown in Figure 4, the replicated data closely follows the patterns of actual perceptual responses, exhibiting consistency across different statistical quantiles.

**Figure 5:**
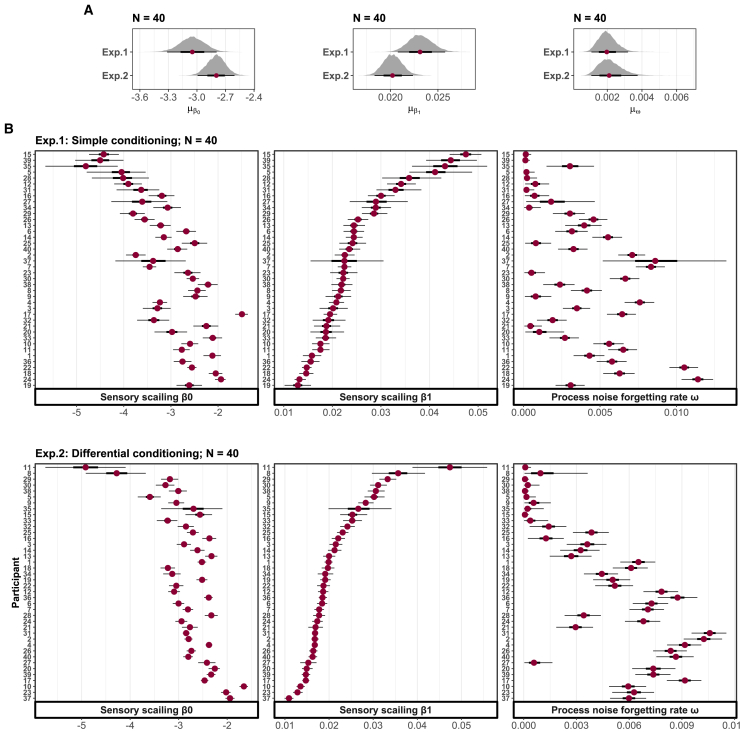
Perceptual model parameter estimates Parameter estimates with 95% credible interval of the three important parameters in the perceptual model. (A) Posterior distributions of the group mean for the scaling parameter $\beta_{0}$, $\beta_{1}$, and *ω*. (B) Posterior distributions of the individual scaling parameter $\beta_{0,i}$, $\beta_{1,i}$, and $\omega_{i}$ (ordered by the 50% quantile of the perceptual slope parameter $\beta_{1,i}$).

With a multilevel structure, the model is capable of estimating these three perceptual parameters both at the group and individual levels, as illustrated in Figure 5. The parameters $\mu_{\beta_{0}}$ (mean of population-level $\beta_{0}$) and $\mu_{\beta_{1}}$ (mean of population-level $\beta_{1}$) encapsulate the group-level information regarding sensory mapping. In Experiment 1 and 2, $\mu_{\beta_{0}}$ displayed a 95% credible interval of [−3.31, −2.79] and [-2.99, −2.61], along with median values of −3.05 and −2.80. On the other hand, $\mu_{\beta_{1}}$, in two experiments, displayed a 95% credible interval of [0.021, 0.026] and [0.018, 0.022], and the median values of 0.023 and 0.020. This parameter governs the overall steepness of the sensory mapping function. A larger value leads to a more pronounced and rapid change in the mapping process, making the function steeper around the midpoint of the logistic curve.

**Figure 6:**
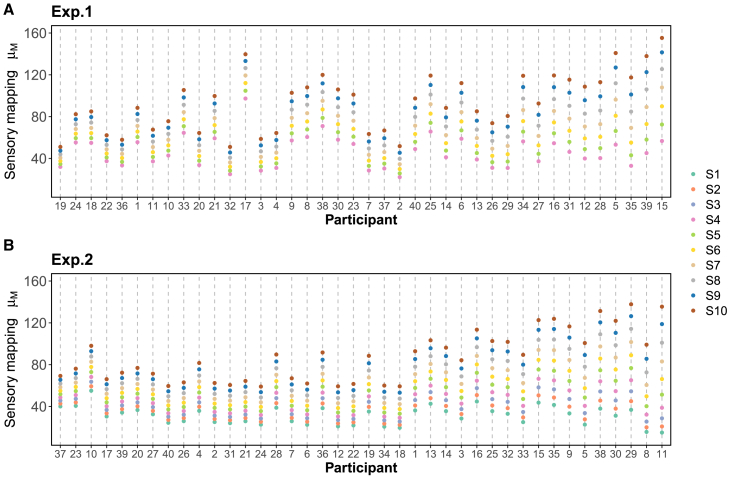
The sensory mapping patterns For each physical stimulus, individual participants demonstrate a distinct mapping from physical to perceptual quantities. The mental representation of each experimental stimulus is calculated with the sensory mapping scaling parameters $\beta_{0}$ and $\beta_{1}$ (50th quantile from the MCMC samples, ordered by the 50% quantile of the perceptual slope parameter $\beta_{1,i}$). (A) and (B) display the mapping patterns for Experiment 1 (simple conditioning) and Experiment 2 (differential conditioning), respectively.

In the panel b of Figure 6, a diverse range of inter-individual variations becomes evident through the broad spectrum of $\beta_{0,i}$ and $\beta_{1,i}$ estimations. These variations give rise to unique patterns in how participants map physical stimuli to mental representations, as shown in Figure 6. Across both experiments, certain participants exhibit lower sensitivity to alterations in physical quantity, resulting in perceptual responses that are generally quite similar across different stimuli. Consequently, these participants are more likely to encounter confusion between different stimuli, given that minor perpetual uncertainties can already lead to overlapping mental representations. Conversely, another group of participants demonstrates heightened sensitivity to shifts in physical quantity, leading them to respond more distinctly to different stimuli and necessitating a greater degree of perceptual uncertainty to induce overlapping perceptual distributions.

#### Dynamic perception

Within each trial, prior perceptual expectations are combined with new sensory input to form the current perception. This integration is governed by the Kalman gain ($\alpha_{\phi }$), which determines the relative importance of prior expectations versus new sensory information. In essence, the Kalman gain adjusts the balance between relying on past experiences and incorporating fresh evidence. A low Kalman gain means prior expectations dominate perception when new sensory input is less reliable. Conversely, a high Kalman gain gives more weight to new input, shaping perception accordingly. This dynamic adjustment balances past and present information based on their relative reliability.

**Figure 7:**
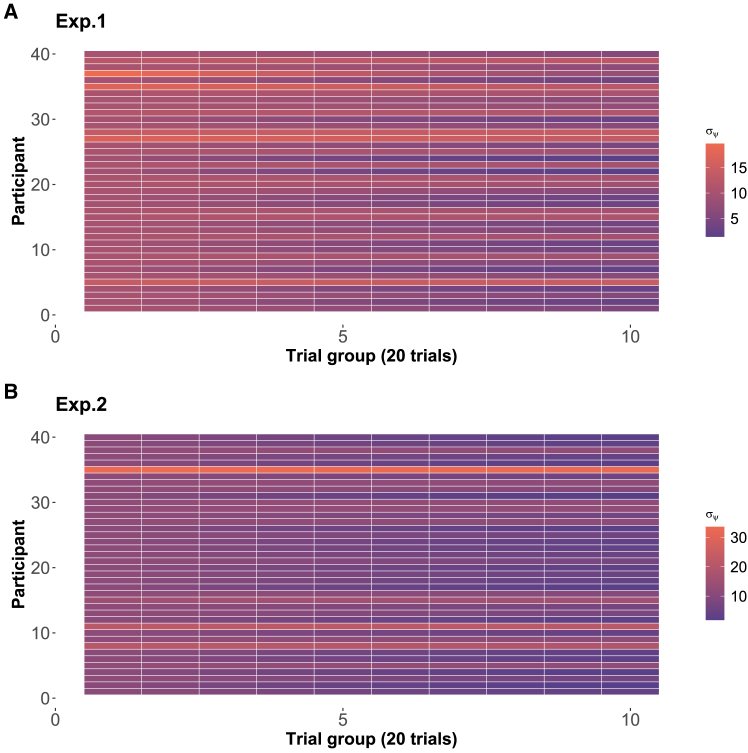
Perceptual uncertainty The two panels illustrate the mean of perceptual uncertainty (from 50th quantile of MCMC samples) for every 20 trials in Experiment 1 (A) and Experiment 2 (B).

The model captures how perceptual uncertainty evolves dynamically over time through two key parameters. The first is a decay rate $\omega_{i}$ that governs how quickly an individual’s perceptual uncertainty changes with repeated stimulus exposure. At the population level, this decay rate $\mu_{\omega }$ showed similar estimates across both experiments, with 95% credible intervals of [0.001,0.003] and [0.001,0.004] in Experiments 1 and 2 respectively, both having median values of 0.002. The second parameter *η* represents the initial magnitude of perceptual uncertainty at the group level, with 95% credible intervals of [10.63, 11.46] and [11.03, 12.34] for the two experiments, and median values of 11.04 and 11.68 respectively. Figure 7 illustrates how this perceptual uncertainty evolves throughout the experiments. Most participants showed a gradual decrease in uncertainty over time, indicating that repeated exposure to stimuli leads to more precise and stable perceptual representations.

The model also includes a sensory mapping uncertainty parameter $\sigma_{S,i}$ that captures individual differences in how consistently people perceive physical stimuli. This parameter reflects the stability of an individual’s perceptual system - a lower value indicates more consistent perception of the same physical stimulus across repeated presentations. The majority of participants in both experiments demonstrated relatively stable perception with $\sigma_{S,i}$ values below 1. This perceptual stability influenced how individuals weighted new sensory information versus their prior perceptual experience, as measured by the Kalman gain which remained above 0.5 for most participants throughout the experiments (Method S2: Kalman Gain Analysis and Figure S2). The consistently high Kalman gain suggests that participants maintained more sensory-based perception of the stimuli, integrating some influence from past perceptual experiences without converging on them too much.

### Generalization

Upon scrutinizing the perceptual patterns evident in both experiments using the state-space perceptual model, our focus shifted to assessing how these different mental stimulus representations contributed to the observed generalization behavior. To accomplish this, we ran the computational model of generalization^3^ twice. One run utilized a point-based approach where stimuli are considered as points in a mental space with their coordinates directly derived from perceptual data (Figure 1B). Here, stimulus similarity is derived by the Euclidean distance between coordinates, thereby ignoring the influence of perceptual uncertainty. In the new model, stimuli are represented as probability distributions that emerged from a Bayesian perception process, with now stimulus similarity being reflected in the overlap between two stimulus distributions (Figure 1C).

The generalization model integrates error-driven learning^39^ and similarity-based generalization processes.^2^ Through repeated exposures to the learned stimulus, stimulus-outcome associations are updated and decay exponentially with decreasing stimulus similarity. Depending on the model, this similarity is derived from point distances or by the overlap in perceptual distributions. To capture distinct patterns of generalization behavior, the model employs a mixture structure with four pathways: Non-Learners and Overgeneralizers represent maladaptive generalization patterns characterized by learning deficits or excessive generalization, respectively. Physical Generalizers and Perceptual Generalizers differentiate whether perceptual variability impacts generalization patterns (Perceptual Generalizers; Figures 1B and 1C) or not (Physical Generalizers; Figure 1A).

#### Model comparison

To assess the relative data fit, we integrated the two perceptual assumptions into a single model (referred to as a super model). Within this framework, we estimated the model selection parameter $\beta_{M}$, which provides information on which sub-assumption (model) better fits the data, and computed the Bayes factor based on the estimations of $\beta_{M}$.

**Figure 8:**
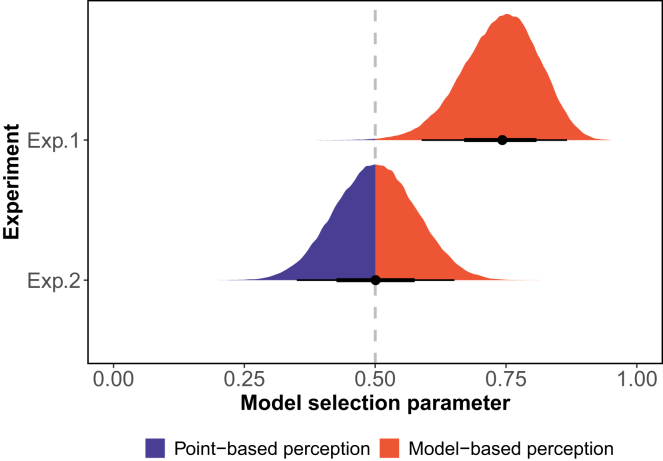
Posterior distributions of the model selection parameter The two models, each based on different assumptions about perceptual distances, are combined into a single super model. The likelihoods of both models are integrated in a linear manner, with a model selection parameter denoted as $\beta_{M}$. The prior distribution for $\beta_{M}$ is specified as Uniform(1,1). The figure displays the posterior distribution of $\beta_{M}$, each comprising 120,000 samples. Values greater than 0.5 are indicated with the red color, suggesting that the observed data show a preference for the model-based perceptual assumption. Conversely, values less than 0.5 are shown in blue color, indicating that the observed data exhibit a preference toward the descriptive perceptual assumption.

Examining the posterior samples of $\beta_{M}$ as depicted in Figure 8 and computed Bayes factors, we observe a distinct preference direction in Experiment 1. The data strongly favored the perceptual distributions model. In Experiment 1, the posterior distribution of $\beta_{M}$ exhibited a 95% confidence interval of [0.59, 0.87], with a median value of 0.74 and a BF = 18.833. On the other hand, in Experiment 2, the data did not show a clear preference for either perceptual assumption, suggesting a more balanced performance between the model with two distinct perceptual assumptions in that context (i.e., the posterior distribution of $\beta_{M}$ had a 95% confidence interval of [0.35, 0.65], with a median value of 0.50 and a BF = 0.197).

#### Latent group allocation

**Figure 9:**
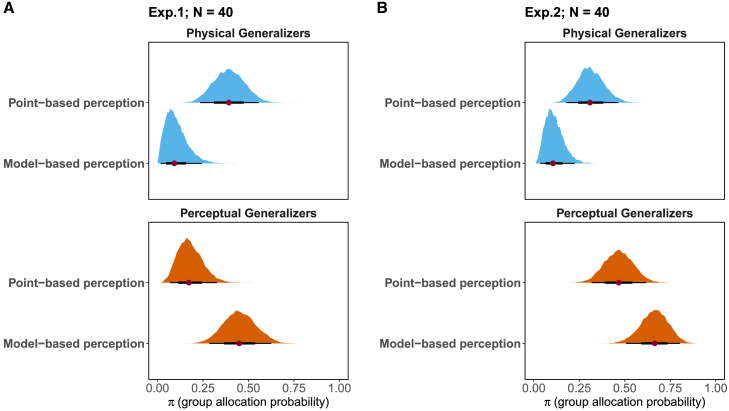
Posterior MCMC samples of group allocation parameter The proportion of the posterior MCMC samples of the group allocation parameter in Experiment 1 (A) and 2 (B). The point distance distribution depicts the posterior samples when the model adopts descriptive perceptual patterns. In this approach, the current perception is calculated as the absolute difference between the current perceptual response and the cumulative average of the perceptual response to the conditioned stimulus. On the other hand, the modeled distance distribution illustrates the posterior samples when the model incorporates model-based perceptual patterns, integrating probabilistic and dynamical assumptions of perception.

Next, we compared if the latent group allocation was affected by the different ways of stimulus representations. In Figure 9, it is shown that the use of stimulus distribution resulted in higher probability of having Perceptual Generalizers in both experiments (Experiment 1: 95% CI[0.28,0.63], median = 0.45; Experiment 2: 95% CI[0.51,0.80], median = 0.66) compared to the adoption of perceptual distance based on point estimates (Experiment 1: 95% CI[0.07,0.33], median = 0.17; Experiment 2: 95% CI[0.32,0.62], median = 0.47). An opposite pattern can be observed for the probability of Physical Generalizers allocation, with lower posterior probability in the new model (Experiment 1: 95% CI[0.02,0.24], median = 0.09; Experiment 2: 95% CI[0.03,0.23], median = 0.11) compared with the point-based approach (Experiment 1: 95% CI[0.23,0.56], median = 0.39; Experiment 2: 95% CI[0.18,0.46], median = 0.31). As to the probability of Non-Learners and Overgeneralizers allocation, as expected, it remains nearly identical regardless of the particular perceptual assumption (Method S3: Group Allocation Patterns and Figures S3 and S4).

## Discussion

The current study presents a framework for comparing two distinct approaches to modeling how humans mentally represent stimuli and its impact on fear generalization responding. The first approach, established in previous research,^3^ acknowledges that perception deviates from physical reality but represents these perceptual differences as discrete points in mental space. Our new approach extends this by conceptualizing perception as probability distributions that emerge from the continuous interaction between sensory evidence and prior expectations. This shift from point-based (Figure 1B) to distribution-based (Figure 1C) representations captures additional complexities of human perception: the inherent uncertainty in perceptual processing, the individual difference in perceptual processing, and the dynamic evolution of distributional perception over time. By comparing these approaches, we demonstrate how incorporating probabilistic representations provides new insights into how perceptual processes shape fear generalization behavior.

Through a comparative analysis of the same generalization model utilizing distinct stimulus representations, we contrasted two fundamental approaches to quantifying perceptual similarity. Our model comparison revealed distinct patterns across the two experiments. In Experiment 1, we found strong evidence favoring the distribution-based over point-based perceptual similarity (BF=18.833). Importantly, both approaches substantially outperformed models assuming veridical stimulus perception (Physical Generalizers). In Experiment 2, while the model comparison showed no clear preference between point-based and distribution-based approaches (BF=0.197), both again proved superior to veridical perception models. The distribution-based approach led to a marked increase in the proportion of participants classified as Perceptual Generalizers in both experiments - from a median of 17%–45% in Experiment 1, and from 47% to 66% in Experiment 2. Correspondingly, we observed a decrease in Physical Generalizers (Experiment 1: from 39% to 9%; Experiment 2: from 31% to 11%). This consistent shift in group allocation across experiments, even when overall model performance was equivalent, suggests that modeling perceptual uncertainty reveals important individual differences in how perception shapes generalization. The different patterns of model preference between experiments might be attributed to several factors: the increased attention demands of differential conditioning could have led to more consistent perceptual responses, reducing the importance of modeling uncertainty, or participants might have exhibited greater variability in how they approached the differential conditioning task. Future research should systematically investigate these potential explanations to better understand how task demands shape the relationship between perceptual processes and generalization behavior, particularly focusing on when and why explicitly modeling perceptual uncertainty becomes crucial for understanding generalization patterns.

In the process of transitioning from learning associations within a specific context to applying that learning in new situations, humans continually engage with physical inputs. Yet, the findings within the realm of perception,^17^^,^^18^^,^^19^^,^^20^ in conjunction with our current findings, make it evident that humans do not possess flawless perception. Instead, human perception operates through a non-linear sensory mapping, and individuals perceive the physical reality through the lens of inherent perceptual uncertainties. Regrettably, current theories of stimulus representation in learning and generalization, such as elemental representation models^22^^,^^23^ or neural network models,^24^ all presume that stimulus representation maps solely to the physical dimension. This assumption suggests that more similar physical features result in greater overlaps in the distribution of stimulus representations, ultimately falling short in capturing the idiosyncratic and dynamic nature of physical-to-perception mappings. The multidimensional scaling method,^2^ while acknowledging the mapping from physical to perceptual space, assumes that perception for a stimulus is a fixed point in mental space, remaining invariant over time or individuals. This perspective, treating perception as a deterministic system, overlooks the inferential and probabilistic nature inherent in perception.

In this work, we advance the understanding of generalization by modeling perception as dynamic probability distributions rather than fixed points in mental space. These distributions capture three fundamental aspects of human perception: how individuals process incoming sensory information, how they integrate this information with past experiences, and how confident they are in their perceptions. When determining whether learning should transfer between stimuli, our framework examines the overlap between their perceptual distributions - a high degree of overlap suggests strong similarity, while minimal overlap indicates distinct percepts. This probabilistic approach reveals subtle but important phenomena that point-based models miss. For instance, two stimuli might be perceived quite similarly on average, yet rarely trigger generalization if people perceive them with high certainty, creating narrow distributions with minimal overlap. Conversely, even when average perceptions differ moderately, uncertain perception of both stimuli can create a focused region of distributional overlap, promoting consistent generalization. By capturing both the content of perception (where distributions are centered) and its quality (how precise they are), our framework provides new insights into why the same physical similarities between stimuli can lead to markedly different patterns of generalization across individuals and contexts.

Generalization holds a significant theoretical foundation not only within other cognitive domains such as learning^40^^,^^41^ and memory,^42^^,^^43^ it constitutes a foundational cognitive process that underpins a range of behaviors, including but not limited to categorization,^44^ motor learning,^45^ language processing,^46^ and face recognition.^47^ This serves to accentuate its pivotal role within the domain of cognitive science. While the significance of variability in the context of learning and generalization has been extensively deliberated,^48^ the bulk of this discourse has predominantly centered around the physical domain. Yet, the exploration of variability within the human perceptual system and its consequential impact on relevant behavior remains relatively understudied. The increased proportion of individuals allocated to the Perceptual Generalizer group through the probabilistic perceptual similarity underscores the role of internal variability within the perceptual system in shaping observed generalization behavior.

The importance of perceptual variability, or more broadly, the intricacies of the perceptual process, also extends beyond theoretical implications to encompass clinical perspectives. Aberrant generalization has garnered empirical attention as a potential driving factor in anxiety disorders.^25^^,^^26^^,^^27^ Moreover, evidence indicates that individuals with anxiety disorders exhibit poorer stimulus identification abilities compared to their healthy counterparts.^29^ However, the causal direction between anxiety disorder symptoms and stimulus identification remains an open empirical question. Furthermore, the association between dynamic and probabilistic perceptual processes and clinical disorder symptoms remains largely unexplored, although there are emerging reports of altered perceptual inference processes in certain patient populations or personality traits.^21^ In this context, the utilization of the current computational model not only provides insights into the associations between the generalization behavior of an individual and the underlying perceptual process but also into the dynamics through which the perceptual process engenders such behavior. For instance, individuals exhibiting a gradual decrease in Kalman gain over time tend to display an increasing bias toward previous perceptual experiences, a phenomenon with a profound impact observed in several mental disorders, contributing significantly to the overall shaping of perceptual patterns.^49^^,^^50^^,^^51^

Additionally, the perceptual sensitivity to different stimulus might also provide valuable insights, an example for this can be found in a recent study in which stimulus-based auditory perceptual plasticity is found to explain the overgeneralization behavior of the generalized anxiety disorder (GAD) patients.^29^ Examining how diverse perceptual processes shape generalization behavior can provide clinicians with valuable insights not only into the extent to which problematic generalization behavior is influenced by biased perception but also into the workings of inherent perceptual processes that contribute to the eventual manifestation of such behavior. In sum, the knowledge of inherent perceptual process may offer clinicians a deeper understanding of the underlying mechanisms involved in anxiety disorders and aid in developing more targeted therapeutic interventions.^52^

### Limitations of the study

Currently, there is no unified and coherent assumption regarding the optimality of human perception within the Bayesian framework.^15^ Moreover, a consensus theory on how perceptual prior should be formulated remains elusive. Some studies have adopted a fixed perceptual prior distribution to represent a macro belief about the physical world,^17^^,^^19^ while others have embraced a more experience-driven perceptual prior distribution.^18^^,^^53^ In our study, we adopted a simple assumption for formulating the perceptual prior, considering past perceptual experiences as the sole prior for the current perceptual system. However, future research can delve deeper into the formulation of perceptual priors. For instance, in the context of fear generalization, the conditioned stimulus often carries more salient experiences, such as fear or pain. A pertinent question arises regarding whether these highly salient experiences contribute to the formulation of a macro perceptual prior that governs the dynamic updating process of perception. Further exploration and refinement of perceptual prior formulations could offer valuable insights into the complex interactions between perceptual experiences and the updating of perceptual systems. By addressing these issues, we can advance our understanding of how human perception adapts to various contexts and experiences, paving the way for more comprehensive models of perceptual processes and their implications for generalization behaviors. Moreover, delving deeper into the investigation of the perceptual prior can also aid in comprehending the influence of fear learning on the modification of the perceptual system,^54^ particularly considering the lack of consistent empirical behavioral findings in this domain.^55^

Another limitation of the current study pertains to its exclusive reliance on self-report responses to assess fear learning, perceptual, and fear generalization processes. While there is some evidence suggesting a strong correlation between self-report responses and physiological or neuronal measures in fear learning, an ongoing debate remains regarding the extent to which we can achieve a consensus on fear-related behavior^56^^,^^57^ through diverse response channels.^58^^,^^59^ This unsolved debate can also apply to the field of perception.^60^ Therefore, we may expect a more complicated relationship when we attempt to investigate both processes at the same time. Previous empirical studies have demonstrated the impact of perceptual variability on generalization with startle eye-blink responses^31^—a physiological manifestation of the body’s automatic reaction to sudden stimuli. The findings of our study hold potential for broader generalizability with such a physiological measure. Furthermore, there is a need for further exploration of the generalizability of the modeling findings to different types of learning paradigms, stimuli with elevated levels of complexity, and multi-dimensional features.

In the present study, the determination of similarity between the currently encountered stimulus and the previously learned stimulus relies solely on perceptual data. However, this approach assumes a precise alignment between the perceptual memory associated with the learned stimulus and the perceptual encoding captured during the initial encounter. Regrettably, this assumption overlooks the potential influence of memory processes, which can exert a significant impact on both the encoding and retrieval of memories. Inter-individual and intra-individual differences during encoding, retrieval, or mechanisms acting on these processes may introduce substantial biases in memory that can deviate considerably from the objective representation of stimuli.^61^^,^^62^ Consequently, the exclusive reliance on perceptual data to determine perceptual distance may fall short in capturing the complexities and variations introduced by memory processes. This situation calls for further investigation and consideration in the study of generalization behaviors. In future studies, it is encouraged to develop models that incorporate recent theories of human memory and collect memory data throughout generalization testing.^63^ This approach would provide a more accurate representation of how individuals perceive and memorize the physical world, thereby advancing our understanding of the intricate interplay between perceptual and memory processes and their combined impact on human generalization behavior.

### Conclusion

Human behavior is a multifaceted phenomenon frequently involving multiple psychological processes and mechanisms. Generalization, as one such behavior, manifests observable traits that can emerge from distinct mechanisms. The comprehension of the congruent relationship between diverse cognitive mechanisms and generalization behavior necessitates a deeper understanding. In this research, we formulate a modeling framework to integrate perceptual mechanisms characterized by their probabilistic and dynamic nature into the process of generalization. This framework enables us to perceive generalization behavior as an outcome arising from the intricate interplay among learning, perception, and generalization mechanisms. Consequently, it affords us a more profound insight into how humans generalize, not merely due to an inherent inclination to transfer previous learning but also owing to the profound interaction between cognition and perception of physical reality. Ultimately, this integrated approach may contribute to the refinement of generalization theories and advance our knowledge of the fundamental mechanisms that shape human generalization behavior.

## Resource availability

### Lead contact

Further information and requests for resources should be directed to and will be fulfilled by the Lead Contact, Kenny Yu (kenny.yu@kuleuven.be).

### Materials availability

This study did not generate new data. All data used in this study were from previously published fear conditioning experiments, as described in Yu et al. (2023).^3^

### Data and code availability

• **Data:** The fear conditioning experiment data (total N=80) have been deposited at the Open Science Framework repository as https://doi.org/10.17605/OSF.IO/SXJAK and are publicly available as of the date of publication (see also Yu et al. (2023)^3^).
• **Code:** All original code, including the computational models (perceptual and generalization models) and the scripts for simulations, statistical inference, and analysis (implemented in R 4.1.1 and JAGS 4.3.1), have been deposited at the Open Science Framework repository as https://doi.org/10.17605/OSF.IO/SXJAK and are publicly accessible as of the date of publication.
• **Additional Information:** Supplementary information for the modeling details are available at the Open Science Framework repository as https://doi.org/10.17605/OSF.IO/SXJAK and are publicly accessible as of the date of publication.

## Acknowledgments

K.Y. is supported by an 10.13039/501100003130FWO research project (co-PI: J.Z., G079520N). J.Z. is a Postdoctoral Research Fellow of the Research Foundation Flanders (FWO, 12P8623N) and received funding from the 10.13039/100005156Alexander von Humboldt Stiftung. K.Y. , W.F., and F.T. are also supported in part by the Research Fund of KU Leuven (C14/23/062). The resources and services used in this work were also provided by the VSC (Flemish Supercomputer Center), funded by 10.13039/501100003130FWO and the Flemish Government. The funders had no role in study design, data collection and analysis, decision to publish or preparation of the manuscript.

## Author contributions

Conceptualization, K.Y., W.F., F.T., and J.Z.; methodology, K.Y., W.F., F.T., and J.Z.; software, K.Y.; formal analysis, K.Y.; investigation, K.Y.; resources, K.Y.; data curation, K.Y.; writing – original draft, K.Y.; writing – review and editing, K.Y., W.F., F.T., and J.Z.; visualization, K.Y.; supervision, F.T. and J.Z.; funding acquisition, J.Z.

## Declaration of interests

The authors declare no competing interests.

## STAR★Methods

### Key resources table

**Table:** 

REAGENT or RESOURCE	SOURCE	IDENTIFIER
**Deposited data**		

Fear conditioning experiment data	Open Science Framework (OSF)	https://doi.org/10.17605/OSF.IO/SXJAK

**Software and algorithms**		

Analysis codes	Open Science Framework (OSF)	https://doi.org/10.17605/OSF.IO/SXJAK

**Other**		

Supplementary Information	Open Science Framework (OSF)	https://doi.org/10.17605/OSF.IO/SXJAK

### Experimental model and subject details

No new experimental data were collected for the current study. Instead, we conducted a re-analysis of two previously published datasets from Yu et al. (2023).^3^ Below, we provide a summary of the original experimental procedures and participant details for context.

#### Human participants

The original study included a total of 80 healthy adult participants, with 40 participants in each of two experiments. Both experiments included 40 participants (65% female, 35% male), with mean ages of 22.0 years (SD = 5.3) in Experiment 1 and 24.0 years (SD = 8.9) in Experiment 2. All participants were recruited via KU Leuven’s online experiment management system and received either course credits or monetary compensation (€12 for Experiment 1 and €16 for Experiment 2). The study was approved by KU Leuven’s Social and Societal Ethics Committee (Approval Reference: G-201610641) and all participants provided written informed consent prior to participation. While the current study did not examine potential sex or gender effects on fear generalization, a recent investigation using a comparable experimental paradigm found no gender differences in fear generalization behavior and underlying processes.^34^

In the original study, sample size was determined based on previous studies investigating fear generalization. In Experiment 1 (simple conditioning paradigm), all participants underwent the same experimental procedure. In Experiment 2 (differential conditioning paradigm), participants were counterbalanced in their assignment of CS+ and CS-, where half of the participants received the smallest circle (S1; 50.8 mm) as CS+ and the largest circle (S10; 119.42 mm) as CS-, while the other half received the opposite assignment.

The original experimental procedures received ethical approval from the Social and Societal Ethics Committee at KU Leuven (Approval Reference: G-201610641).

### Method details

This study presents a computational reanalysis of previously published experimental datasets^3^ using a two-step modeling approach that separates perceptual and generalization processes. First, we applied principles from Bayesian perception theory to model participants’ trial-by-trial perceptual judgments, capturing how physical stimuli are transformed into probabilistic mental representations. From this perceptual model, we derived a new perceptual distance metric based on distribution overlaps, which was then incorporated into our previously established generalization model^3^ to predict fear learning responses. This approach allowed us to evaluate how different assumptions about perception influence generalization behavior while maintaining a tractable modeling framework.

Importantly, we did not model generalization itself as a Bayesian process; instead, we applied Bayesian principles only to model perceptual processes, and then used the outputs from this perceptual model to derive distances that served as inputs to our established generalization model.^3^ This two-step approach allowed us to compare how different perceptual assumptions shaped generalization behavior while maintaining a consistent generalization framework. By separating the perceptual modeling from the generalization process, we ensured that the probabilistic nature of our perceptual model did not inadvertently bias our comparison of different perceptual assumptions.

For complete experimental details of the original data collection, see Yu et al. (2023).^3^

#### Experiments

The datasets used in this reanalysis came from two experiments that each comprised 40 participants. In both original experiments, participants underwent an acquisition phase, where they learned the associations between conditioned stimuli (CSs) and an unconditioned stimulus (US), followed by a generalization phase where test stimuli (TSs) were presented. The CSs and TSs were represented by circles with white outlines against a black background. The unconditioned stimulus used in both experiments was a noxious electrocutaneous stimulus.

The overall stimulus set of CSs and TSs comprised ten circles, labelled as S1 to S10, with diameters ranging from 50.80 to 119.42 mm, increasing by 7.624 mm between each step. In Experiment 1, which employed a simple conditioning paradigm, a subset of seven circles (S4 to S10) was utilized. The middle circle (S7; 96.54 mm) served as the CS+, while the remaining six stimuli were designated as TSs. In Experiment 2, a differential conditioning paradigm was employed, utilizing the entire stimulus set (S1 to S10). The assignment of the CS+ and CS- was counterbalanced among participants. The smallest circle (S1; 50.8 mm) and the largest circle (S10; 119.42 mm) alternated between being the CS+ or CS-. The remaining eight stimuli exclusively served as TSs and were solely presented during the generalization phase.

In each trial of both experiments, participants were required to provide ratings on two scales. Firstly, they rated the diameter of the stimulus using a size Visual Analogue Scale (VAS), which ranged from 0 to 200 millimeters. Responses on this task show large inter-individual differences but high across-days reliability within individuals.^35^ Secondly, they rated their expectancy of experiencing an unconditioned stimulus (US) after observing the presented circle using an expectancy VAS, with ratings ranging from ‘no shock’ (1) to ‘definitely a shock’ (10).

In Experiment 1, the acquisition phase consisted of 14 CS+ trials, where the CS+ was paired with the unconditioned stimulus (US) in 7 trials, resulting in a reinforcement rate of 50%. In Experiment 2, the acquisition phase included 12 CS+ trials and 12 CS- trials. Notably, 83% of the CS+ trials were paired with the US, while the CS- trials were never paired with the US. The generalization phase in Experiment 1 encompassed four blocks, whereas Experiment 2 comprised three blocks, with each block being separated by a 3-minute break. Importantly, the US was never paired with the CS- or the TS trials; it was exclusively associated with the CS+ trials in both experiments. Each block in Experiment 1 consisted of 22 CS+ trials and 24 TS trials, and to prevent the extinction of the conditioned response, each block was always initiated with ten consecutive CS+ trials, known as re-acquisition. On the other hand, Experiment 2 incorporated 14 CS+ trials, 8 CS- trials, and 32 TS trials in each block, with each block commencing with six consecutive CS+ trials. Past research successfully used CS+ re-acquisition and large number of stimulus repetitions within a context of fear generalization.^35^^,^^55^

#### Computational model of generalization

Our computational framework advances the quantification of perceptual influences on fear generalization in two key ways (see Figure S5 for the Directed Acyclic Graph). First, we modeled how individuals perceive stimuli using a state-space model that captures both the probabilistic and dynamic nature of perception. Second, we introduced a new method for calculating stimulus distances based on the overlap between perceptual distributions, replacing our previous approach that relied on direct differences between mental coordinates (based on perceptual ratings).^3^ Throughout our modeling, we employed weakly to non-informative priors given the current uncertainty about underlying processes (Method S4: Prior Specifications for Computational Models and Tables S1 and S2). A prior sensitivity analysis conducted for both the perceptual and generalization models demonstrates that our results are robust to a range of plausible prior choices (Method S5: Sensitivity Analysis of Model Priors and Figures S6 and S7). The following sections detail first the generalization model’s core elements, then our new approach to computing perceptual distances.

#### Model architecture

The computational model incorporates a mixture structure, enabling the generation of four distinct theoretically or clinically relevant behavior paths. The first group, Non-Learners, is characterized by a learning rate of 0, indicating that the error-driven learning process has not occurred, and consequently, there is no expectation to be generalized. The second group, Overgeneralizers, exhibits a very high generalization tendency (indicated by a low value for the generalization rate). This propensity ensures that the similarity remains consistently higher than 70%, even when encountering the most distant stimuli in the experiment, thereby maintaining the US expectation above 70% of the expectation to the learned stimulus regardless of the encountered stimulus.

The remaining two groups, Physical Generalizers and Perceptual Generalizers, presume that individuals have learned the associations between the CS and US to some extent and subsequently generalize these associations to other stimuli (TS) based on similarity. The distinction between these groups lies in the dimension of generalization - physical or perceptual. Physical Generalizers assume no perceptual variability or that perceptual variability does not influence generalization behavior, while Perceptual Generalizers posit a link between perceptual variability and generalization behavior.

The fundamental assumption of the generalization model posits that at each time point, every participant (indexed by i=1,…,n) holds certain expectations regarding US onset denoted as $v_{ij}$ for the conditioned stimulus on each trial (j=1,…,k) in the learning process. In each conditioned stimulus (CS) trial, the associative strengths of the CS(s) and the unconditioned stimulus (US) for individual *i* and trial *j* are updated based on the prediction error, which represents the difference between the current outcome and expectancy, and the individual learning rate ($\alpha_{i}$).^39^ Concurrently, the generalization process formulates the extend of US expectancy transfer ($\lambda_{i}$) to another stimulus $s_{ij}$ by considering mental stimulus distances ($d_{ij}$)^2^ with stimulus coordinates corresponding to either physical stimulus features or perceived stimulus features. Mathematically, this relationship can be expressed in Equations 1 and 2:

(Equation 1) $$v_{ij+1}=v_{ij}+\alpha_{i}\left(r_{ij}-v_{ij}\right)k_{ij}\text{,}$$

and

(Equation 2) $$g_{ij}=v_{ij}e^{-\lambda_{i}d_{ij}}\text{.}$$

where $v_{ij}$ represents the associative strength of the CS(s) in trial *j* for participant *i*, reflecting the time-dependent learned expectation of the CS(s) - US association. In the differential learning experiment, we will distinguish further between $v_{ij}^{+}$ and $v_{ij}^{-}$. Specifically, $v_{ij}^{+}\in \left[0,1\right]$ denotes the associative strength for the CS+ (the excitatory reinforced CS) in the context of the differential learning experiment, while $v_{ij}^{-}\in \left[-1,0\right]$ pertains to the CS- (the inhibitory reinforced CS). The dummy variable $k_{ij}$ is used to control the occurrence of updating, where $k_{ij}\in \{0,1\}$ with 1 indicating updating and 0 otherwise. Its role is to ensure that learning only happens during CS trials. The variable $r_{ij}$ corresponds to the trial outcomes ($r_{ij}\in \{0,1\}$ for the CS+ and $r_{ij}\in \{-1,0\}$ for the CS-). The parameter $\alpha_{i}$ represents the learning rate, regulating the amount of value learning adaptation for individual *i* ($\alpha_{i}\in \left[0,1\right]$), with higher values indicating more learning from the prediction error. The parameter $\lambda_{i}$ is the generalization rate, signifying the rate of decay that ensues at a given fixed value of stimulus distance $d_{ij}$. It concurrently functions as a discriminative factor in identifying Overgeneralizers. An individual is allocated within the Overgeneralizers group if their acquired expectations $v_{ij}$ demonstrate a decay exceeding 70%, even in the presence of the largest stimulus distance.

The mental stimulus distance between the currently encountered stimulus (TS) and the CS(s) is determined by stimulus coordinates, which may correspond to either the physical stimulus features (Physical Generalizers) or the perceived stimulus features (Perceptual Generalizers). Defined as follows:

(Equation 3) $$d_{ij}=|x^{CS}-x_{j}^{TS}|\text{,}$$

where $x^{CS}$ represents the coordinate of the CS, and $x_{j}^{TS}$ signifies the coordinate of the TS on trial *j*, both situated within the physical dimension. In contrast, for Perceptual Generalizers:

(Equation 4) $$d_{ij}=|{\tilde{x}}_{i,1,\ldots ,j}^{CS}-{\tilde{x}}_{ij}^{TS}|\text{.}$$

with ${\tilde{x}}^{CS}i,1,\ldots ,j$ refers to the cumulative mean derived from the repeated presentations of the CS, capturing the perceived features of the CS up to trial *j*, while ${\tilde{x}}_{ij}^{TS}$ specifically denotes the perceived features of the TS at trial *j*.

The integration of learning and generalization processes yields an generalized associative strength, denoted as $g_{ij}$. In the context of differential conditioning, $g_{ij}$ is constrained within the range of [-1, 1], $g_{ij}=v_{ij}^{+}e^{-\lambda_{i}d_{ij}^{+}}+g_{ij}v_{ij}^{-}e^{-\lambda_{i}d_{ij}^{-}}$,^64^ while for simple conditioning, it is limited to [0, 1], $g_{ij}=v_{ij}e^{-\lambda_{i}d_{ij}}$. Notably, the magnitude of $g_{ij}$ reflects the strength of generalized responses - smaller values result in lower generalized responses, while larger values lead to more potent responses. However, it is important to acknowledge that the scale of $g_{ij}$ does not directly align with the scale of observed behavior, which operates on a 1-10 range. This discrepancy necessitates a scale transformation to establish a meaningful relationship between the two. To address this, we adopted a non-linear sigmoid function, which takes into account both the base rate response and scaling parameters. This sigmoid function effectively bridges the gap, mapping the latent generalized associative strength to the observed response in a manner that aligns with the observed behavioral scale. This can be seen in Equation 5:

(Equation 5) $$\theta_{ij}=A+\frac{K-A}{1+e^{-\left(w_{0_{i}}+w_{1_{i}}g_{ij}\right)}}\text{,}$$

The sigmoid function parameters, *A* and *K*, define the lower and upper limits, ensuring that $\theta_{ij}$ aligns with the measurement scale $\theta_{ij}\in \left[1,10\right]$ $y_{ij}\in \left[1,10\right]$ used in this study. Hence, we set A=1 and K=10. $w_{0i}$ represents the baseline response parameter, dictating the response in the absence of CS associative strengths. On the other hand, $w_{1i}$ serves as the scaling parameter, determining the relationship between latent and observed responses.

The final generalization response is assumed to follow a normal distribution with $\theta_{ij}$ being the mean of the distribution:

(Equation 6) $$y_{ij}∼N\left(\theta_{ij},\sigma^{2}\right)\text{.}$$

The parameter *σ* plays a crucial role in regulating the level of response noise, which, in turn, is dependent on the specific group. A defining characteristic of the Non-Learners group is that their final responses are entirely random and unrelated to any learning or generalization processes. To accommodate this behavior, a distinct prior parameterized by *σ* has been assigned specifically for Non-Learners ($m_{i}=1$), setting it apart from the prior applied to the other three latent groups ($m_{i}=2,3,4$).

In the comparison between the generalization model incorporating the point-based perceptual assumption^3^ and the current work’s model-based assumption, it is noteworthy that no changes were anticipated within the Non-Learners and Overgeneralizers groups. These groups inherently exhibit generalization patterns independent of stimulus distance. However, discernable variations were expected within the Physical Generalizers and Perceptual Generalizers groups. The estimates within these groups assess the extent to which generalized responses align more with perceptual stimulus distance than with physical stimulus distance. Should the new perceptual distance variable result in a closer alignment of generalization responses with perceptual distance compared to the perceptual distance in the previous work,^3^ an increase in the number of Perceptual Generalizers and a corresponding decrease in the number of Physical Generalizers are expected. Conversely, if the alignment with perceptual distance is less pronounced, an increase in Physical Generalizers and a decrease in Perceptual Generalizers would be observed.

#### New perceptual stimulus distance

When computing perceptual distance in the generalization process, our previous study^3^ calculated mental inter-stimulus distance $d_{ij}$ as the absolute difference between two point values: the current perceptual response and a memory representation of the CS (computed as the running average of past CS perceptual responses). While computationally straightforward, this approach treated perceptual responses as precise, fixed points in mental space, overlooking two fundamental aspects of human perception: (1) the uncertainty inherent in transforming physical stimuli into mental representations, and (2) the dynamic nature of how current sensory input integrates with prior experiences.

Here, we introduced a theoretically-grounded approach that explicitly incorporates these perceptual processes into distance calculations. With the new operationalization in this current work, distance equates to the amount of distribution overlap (rather than an absolute difference), with CS memory now also conceptualized as a probability distribution. The overlap coefficient provides a method to quantify probabilistic distributions into a deterministic metric, enabling direct comparison with other non-probabilistic assumptions about perceptual distance. This approach not only acknowledges the inherent uncertainty in perception but also allows for a fair and consistent comparison across competing theoretical frameworks. Because the current experimental design does not have information on the memory representation, we postulate that the memory distribution is faithfully encoded during the preceding CS trial and accurately decoded during the subsequent trial. The mean and the standard deviation of perception $\mu_{\psi ,ij}$ and $\sigma_{\psi ,ij}$ and perceptual memory $\mu_{\psi ,ij-1}^{CS}$ and $\sigma_{\psi ,ij-1}^{CS}$ are derived from a perceptual model with the perceptual data.

Crucially, while the concept of response distributions arising from feature overlap has been explored in prior generalization theories,^22^^,^^23^^,^^64^ these frameworks were unable to distinguish whether these distributions stemmed from perceptual uncertainty, memory imprecision, or the generalization process itself. Our current framework addresses this limitation by specifically isolating perceptual processes: we compute distances between stimuli by analyzing the overlap between two distinct distributions - the perceptual distribution of the currently encountered stimulus and the memory distribution of the conditioned stimulus (CS).

The perceptual distance, $d_{ij}$, is calculated using the overlap between two probability densities: $f_{1}\left(x\right)$, representing how the current stimulus is being perceived, and $f_{2}\left(x\right)$, representing how the CS is remembered:

(Equation 7) $$d_{ij}=1-\int \min\left(f_{1}\left(x\right),f_{2}\left(x\right)\right)\;dx\text{,}$$

where

(Equation 8) $$f_{1}\left(x\right)=\frac{1}{\sigma_{\psi ,ij}\sqrt{2\pi }}\;\exp\;\left(-\frac{{\left(x-\mu_{\psi ,ij}\right)}^{2}}{2\sigma_{\psi ,ij}^{2}}\right)\text{,}$$

and

(Equation 9) $$f_{2}\left(x\right)=\frac{1}{\sigma_{\psi ,ij-1}^{CS}\sqrt{2\pi }}\;\exp\;\left(-\frac{{\left(x-\mu_{\psi ,ij-1}^{CS}\right)}^{2}}{2{\left(\sigma_{\psi ,ij-1}^{CS}\right)}^{2}}\right)\text{.}$$

This overlap-based approach offers several advantages. First, it accounts for the probabilistic nature of human perception, acknowledging that perceptual responses are not fixed but arise from distributions with inherent variability. Second, it provides a more comprehensive measure of similarity by incorporating both the central tendencies and uncertainties of the perceptual and memory distributions. When the two distributions perfectly align (i.e., maximal overlap), the perceptual distance is $d_{ij}=0$, indicating high similarity. Conversely, minimal overlap results in $d_{ij}$ approaching 1, signifying low similarity. By adopting this method, we ensure that perceptual uncertainty is explicitly considered, enabling a more nuanced understanding of generalization processes. Moreover, this metric bridges probabilistic and deterministic frameworks, facilitating direct comparison with traditional, non-probabilistic assumptions.

The determination of overlapping regions between the perceptual ($N\left(\mu_{\psi ,ij},\sigma_{\psi ,ij}^{2}\right)$) and memory ($N\left(\mu_{\psi ,ij-1}^{CS},\sigma_{\psi ,ij-1}^{CS^{2}}\right)$) distributions involves calculating the integral that captures the minimum of their respective probability density functions (PDFs) across the entire range, as shown in Equations 7, 8, and 9. To numerically approximate this integral, we employed the Monte Carlo method. By generating a set of random points within the defined range, we estimated the proportion of points that fell within the overlapping region, thereby approximating the corresponding area.

The Monte Carlo implementation involved sampling points uniformly within the range of interest. Specifically, for each randomly generated point, its *y*-coordinate was drawn from a uniform distribution between zero and the maximum value of the corresponding PDF. By comparing the *y*-coordinate of each point with the PDF values, we identified the points residing within the overlapping region and estimated the overlap area.

To avoid increasing the complexity of the generalization model, we adopted a two-step approach. First, we used the perceptual model to fit the size estimation data, yielding perception estimates grounded in the model. Next, we calculated the trial-by-trial perceptual distances using these estimates, resulting in a distance matrix ${\hat{d}}_{ij}$. These values were then integrated into the generalization model. This two-step methodology provides several advantages. It enables a direct comparison of generalization models with different perceptual assumptions while maintaining a consistent model structure. Additionally, it minimizes the risk of one process (i.e., perception or generalization) being mis-specified and subsequently influencing the inference of the other process, ensuring a more robust and interpretable modeling framework.

#### Perceptual model

Our perceptual model captures how humans transform physical stimuli into mental representations through a dynamic process that combines current sensory input with past experiences. We implemented this using a one-dimensional state-space model based on Bayesian principles, where perception emerges from the continuous interaction between incoming sensory evidence and prior expectations. The model’s updating mechanism follows Kalman filter dynamics,^65^ which provides a mathematically principled way to integrate new information with existing beliefs while accounting for their respective uncertainties. This approach has been implemented before to investigate the dynamic patterns of human perception.^20^ The model architecture comprises three fundamental components:

(1) Sensory input processing:

First, physical stimuli are transformed into initial perceptual estimates through a flexible sigmoid mapping function:

(Equation 10) $$\mu_{S_{ij}}=\frac{\Omega }{1+e^{-\left(\beta_{0,i}+\beta_{1,i}x_{s\left[ij\right]}\right)}}\text{,}$$

(Equation 11) $$\psi_{S,ij}∼N\left(\mu_{S,ij},\sigma_{S,i}^{2}\right)\text{.}$$

where Ω=200 represents our rating scale’s upper limit, and $\beta_{0,i}$ and $\beta_{1,i}$ are individual-specific parameters capturing personal differences in the perceptual response. Crucially, this initial mapping produces not a single value but a probability distribution, reflecting inherent sensory uncertainty. The sigmoid function offers distinct advantages over traditional psychophysical functions like Weber’s law or Stevens’ power law for our modeling purposes. While traditional psychophysical functions are derived from averaged data and presuppose uniform perception across individuals, the sigmoid function’s mathematical properties afford greater flexibility in capturing individual differences. The dual scaling parameters enable the accommodation of diverse perceptual mapping patterns (Figure 3A), making it particularly suitable for modeling inter-individual variability in stimulus perception.

(2) Dynamic integration:

The heart of our model is a Kalman filter mechanism that governs how current sensory input is integrated with prior perceptual expectations. This integration process unfolds continuously over time, with each moment requiring the brain to combine new sensory evidence with accumulated prior expectations. At any given time point (j+1), the brain receives fresh sensory evidence ($\mu_{S,ij+1}$) about the current stimulus while maintaining its prior expectation ($\mu_{\psi ,ij}$) from previous experiences. The Kalman filter determines the optimal way to combine these information sources based on their relative uncertainties. The updated perception is computed through a weighted averaging process:

(Equation 12) $$\mu_{\psi ,ij+1}=\mu_{\psi ,ij}+\alpha_{\psi ,ij+1}\left(\mu_{S,ij+1}-\mu_{\psi ,ij}\right)\text{,}$$

This equation illustrates how perception is updated in response to new perceptual experiences. Specifically, it reflects the difference between the expected perception ($\mu_{\psi ,ij}$) and the actual sensory input ($\mu_{S,ij+1}$). The extent of this adjustment is governed by the Kalman gain ($\alpha_{\psi ,ij+1}$), which is defined as:

(Equation 13) $$\alpha_{\psi ,ij+1}=\frac{\sigma_{\psi ,ij}}{\sigma_{\psi ,ij}+\sigma_{S,i}}\text{,}$$

The Kalman gain serves as a dynamic arbiter between new sensory information and prior expectations. When uncertainty in prior expectations ($\sigma_{\psi ,ij}$) is high relative to sensory uncertainty ($\sigma_{S,i}$), the gain approaches 1, causing perception to rely more heavily on new sensory input. Conversely, when prior uncertainty is low relative to sensory uncertainty, the gain approaches 0, leading to greater reliance on prior expectations. This adaptive weighting ensures optimal integration based on the reliability of each information source. Consider how this plays out in practice: When someone has developed very stable prior expectations through repeated exposure to similar stimuli, their prior uncertainty ($\sigma_{\psi ,ij}$) becomes relatively low. If they then encounter these stimuli in a noisy environment (high $\sigma_{S,i}$), the Kalman gain will be small, causing them to rely more on their well-established prior expectations than on the uncertain sensory input. However, if they encounter something unexpected that challenges their prior expectations, or if the sensory evidence is particularly clear and reliable, the weighting will shift to favor the new information.

(3) Uncertainty evolution:

The evolution of perceptual uncertainty represents another crucial dynamic aspect of our model. As individuals accumulate perceptual experiences, the precision of their perceptual estimates changes systematically. This evolution follows a principled updating rule:

(Equation 14) $$\sigma_{\psi ,ij+1}=\left(1-\alpha_{\psi ,ij+1}\right)\sigma_{\psi ,ij}+\eta e^{-\omega_{i}\cdot j}\text{.}$$

The first term, $\left(1-\alpha_{\psi ,ij+1}\right)\sigma_{\psi ,ij}$, reflects how uncertainty naturally decreases as we gain more experience with a stimulus. However, perception typically maintains some degree of flexibility rather than becoming entirely rigid or deterministic. The second term, $\eta e^{-\omega_{i}\cdot j}$, introduces process noise that decays over time at an individual-specific rate $\omega_{i}$. This decay term allows for individual differences in how people balance stability with flexibility in their perceptual systems. The parameter *η* sets the initial magnitude of this uncertainty, while $\omega_{i}$ determines how quickly an individual’s perceptual system stabilizes (Figure 3B).

In summary, our perceptual model captures how sensory information is processed and integrated over time. At each temporal interval, the sensory system transforms physical stimuli into an initial distribution $N\left(\mu_{S,ij+1},\sigma_{S,i}^{2}\right)$. This sensory information is then integrated with prior expectations based on previous experiences, represented by $N\left(\mu_{\psi ,ij},\sigma_{\psi ,ij}^{2}\right)$, to generate the current perceptual distribution $N\left(\mu_{\psi ,ij+1},\sigma_{\psi ,ij+1}^{2}\right)$. The integration process reflects both immediate sensory transformations and accumulated perceptual history, with the relative influence of each determined by the Kalman gain $\alpha_{\psi ,ij}$.

This formulation naturally accommodates individual differences in perceptual processing. For some individuals, the weight given to prior perceptual experiences strengthens over time, manifested through a decreasing trajectory of $\alpha_{\psi ,ij}$ as experiences accumulate. In contrast, others maintain a more flexible perceptual system where current sensory information continues to play a prominent role, evidenced by sustained high levels of $\alpha_{\psi ,ij}$ across time. These individual-specific dynamics emerge from the interaction between the Kalman gain mechanism and person-specific parameters governing uncertainty evolution.

### Quantification and statistical analysis

The statistical inference was carried out using Markov Chain Monte Carlo (MCMC) with the Gibbs sampling method implemented through JAGS.^66^ The analysis was conducted in the statistical computing language R,^67^ utilizing the R package jagsUI.^68^ To ensure robust results, four MCMC chains were executed for the two models - the perceptual and the generalization model, each comprising 100,000 iterations. A burn-in period of 75,000 iterations was implemented to discard initial samples, and a thinning factor of 10 was applied, resulting in a total of 10,000 ($\frac{\left(100,000-75,000\right)}{10}\times 4$) retained samples per parameter. Convergence of the MCMC chains was assessed using the $\hat{R}$ statistic based on Gelman and Rubin diagnostics.^69^^,^^70^ The chains were considered to have reached a stabilized state and attained the target distribution when the $\hat{R}$ value value approached, or was close to, 1.

For the comparison of two models with different assumptions about perceptual distances, we further ran the super model which encompasses both model assumptions and estimated the model selection parameter $\beta_{M}$ with the following MCMC rules: Four MCMC chains with 250,000 iterations each. A burn-in period of 100,000 iterations and a thinning factor of 5, resulting in a total of 120,000 ($\frac{\left(250,000-100,000\right)}{10}\times 4$) retained samples for the model selection parameter $\beta_{M}$. It is crucial to emphasize that the two models being compared share identical model structures, encompassing the same parameters, variables, and likelihood functions. The sole distinction lies in the application of different inter-stimulus distance metrics—point differences versus overlap in distributions.

The construction of a super model and the estimation of the model selection parameter $\beta_{M}$ aim to evaluate whether the model’s performance is enhanced when employing a probabilistic perceptual model compared to the previous descriptive approach. To investigate this, we created a nested structure, wherein two models with different perceptual distance assumptions were incorporated into a single model (i.e., a super model), and subsequently, we combined the likelihoods of these two model assumptions in a linear manner.^71^ Specifically, considering the parameter vectors $\theta_{1}$ and $\theta_{2}$ corresponding to the model utilizing the model-based perceptual distance assumption, denoted as $M_{1}$, and the model employing the descriptive perceptual distance assumption, denoted as $M_{2}$, we thoroughly examined the likelihood function of the observed data *D*:

(Equation 15) $$P\left(D|\beta_{M},M_{1},M_{2}\right)=\beta_{M}P\left(D|\theta_{1},M_{1}\right)+\left(1-\beta_{M}\right)P\left(D|\theta_{2},M_{2}\right)\text{.}$$

where $\beta_{M}∼Beta\left(1,1\right)$, and $\beta_{M}>0.5$ infers that $M_{1}$ outperforms $M_{2}$ and vice versa.

With the properly nested model, we can then compute the Bayes factor^72^^,^^73^ with the Savage-Dickey method^74^^,^^75^ based on the parameter $\beta_{M}$ to determine to what extent the performance of the two models are different. For this, we computed Bayes factor given $H_{0}$ having $\beta_{M}$ fixed to 0.5 and $H_{1}$ having $\beta_{M}$ deviated from 0.5:

(Equation 16) $$\frac{P\left(D|H_{0}\right)}{P\left(D|H_{1}\right)}=\frac{P\left(\beta_{M}=0.5|D,H_{1}\right)}{P\left(\beta_{M}=0.5|H_{1}\right)}\text{.}$$

This implies that to compute the Bayes factor, we only need to consider the ratio between the posterior of the parameter $\beta_{M}$ under the more intricate assumption $H_{1}$ , given the observed data *D*, and the prior of $\beta_{M}$ under $H_{1}$ (see Method S6: Savage-Dickey Density Ratio for the proof). When the Bayes factor is greater than 1, it signifies that there is stronger evidence suggesting that $\beta_{M}$ deviates from 0.5, indicating that models $M_{1}$ and $M_{2}$ exhibit distinct performance. Conversely, if the Bayes factor is less than 1, it indicates that the evidence favours $\beta_{M}$ being close to 0.5, suggesting that models $M_{1}$ and $M_{2}$ have similar performance.

Once a determination has been made regarding the presence of enough evidence indicating the superior performance of one model over another, our attention turns to examining the distribution of posterior samples of the model selection parameter $\beta_{M}$ to determine whether the data favors model $M_{1}$ or $M_{2}$.
