# Supplementary material for: The probabilistic and dynamic nature of perception in human generalization behavior
Source: iScience. 2025 Mar 17;28(4):112228. doi: 10.1016/j.isci.2025.112228 (PMC11995087; doi:10.1016/j.isci.2025.112228)
Supplement: Document S1. Figures S1–S7, Tables S1 and S2, and Methods S1–S6 [file mmc1.pdf]

iScience, Volume *28*

Supplemental information

The probabilistic and dynamic nature

# of perception in human generalization behavior

Kenny Yu, Wolf Vanpaemel, Francis Tuerlinckx, and Jonas Zaman

Figure S1: The parameter recovery of the perceptual model

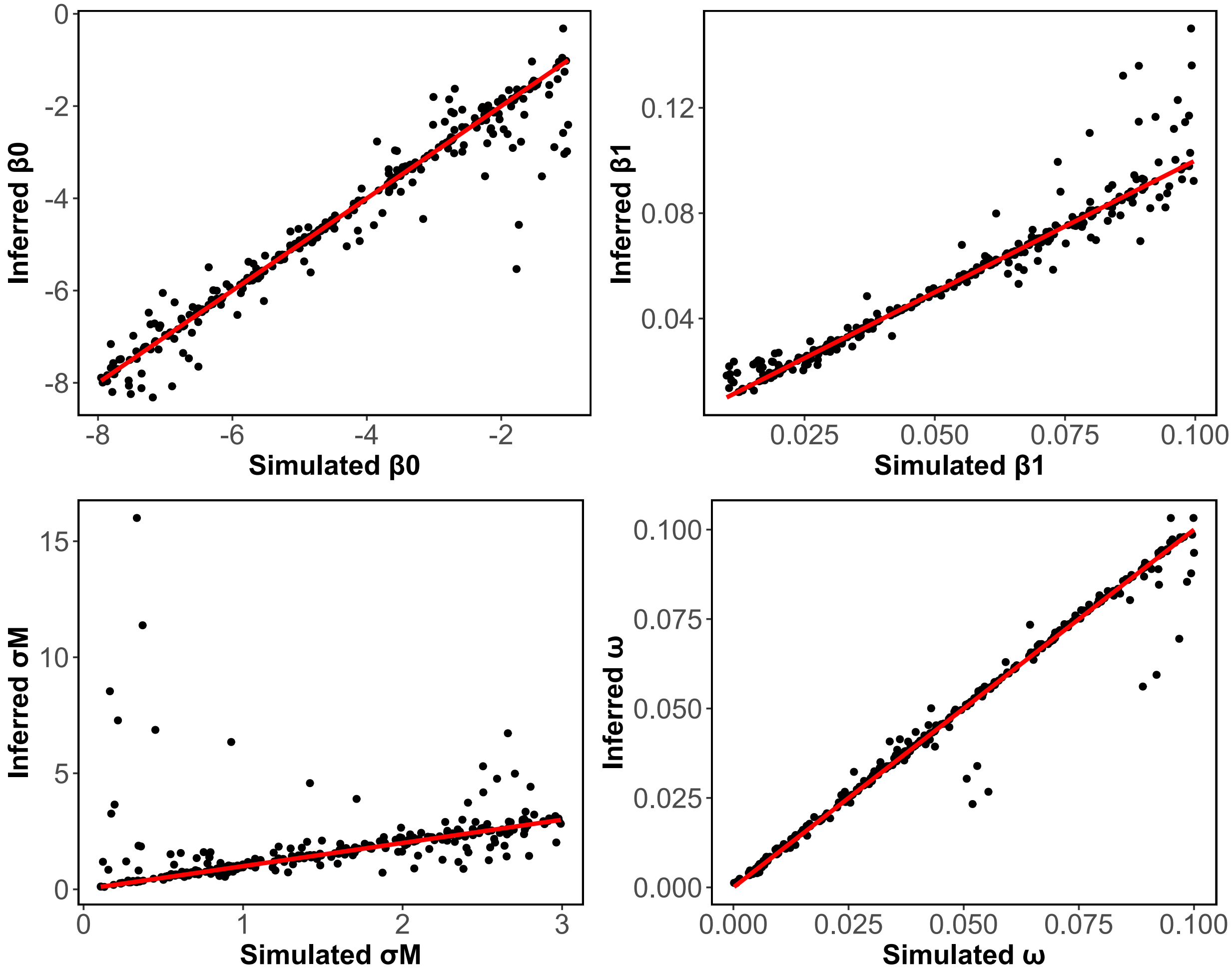

The red straight line represent the perfect recovery.

Figure S2: Patterns of Kalman gain

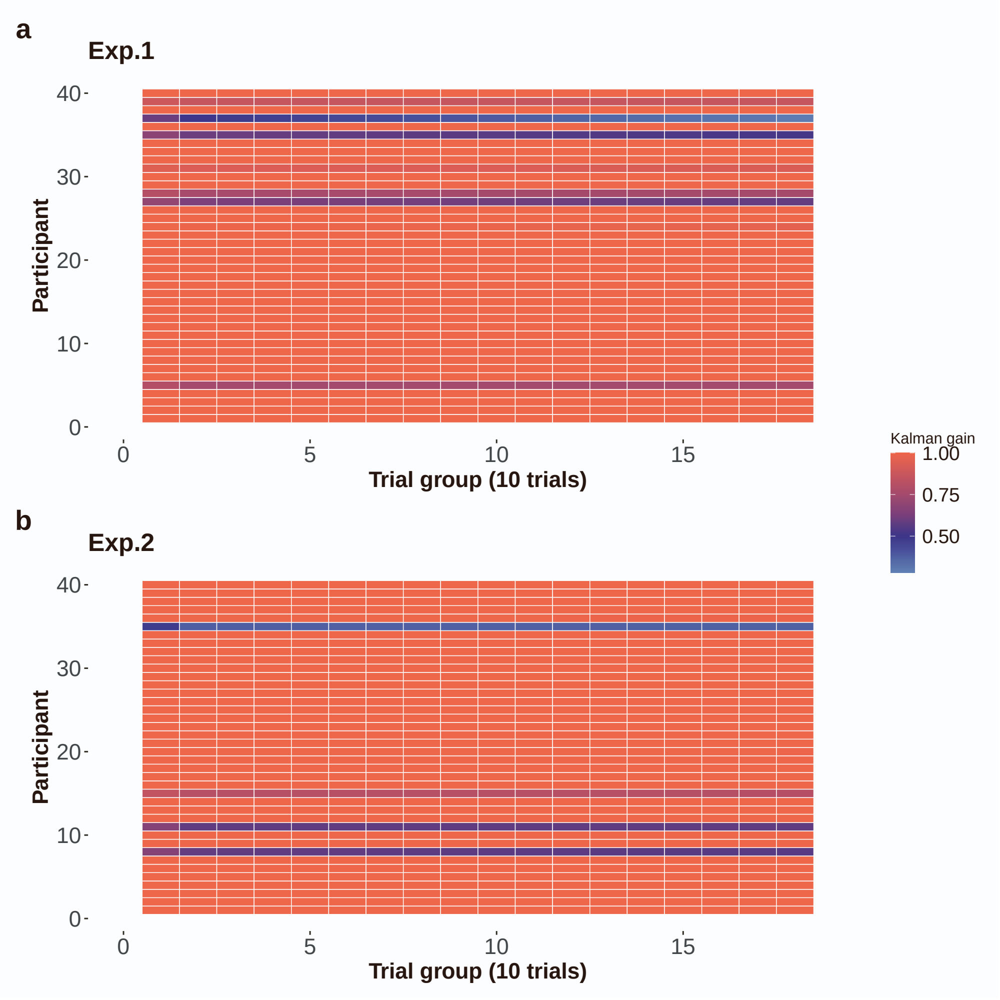

The two panels illustrate the mean of Kalman gain (from 50th quantile of MCMC samples) for every 10 trials in Experiment 1 (Panel a) and Experiment 2 (Panel b). The Kalman gain provides insight into the dynamic nature of perceptual patterns, with higher values indicating a propensity towards the perceptual likelihood distribution, and lower values indicating a preference for relying on previous perceptual experiences.

Figure S3: MCMC samples on the group allocation parameter with the previous model

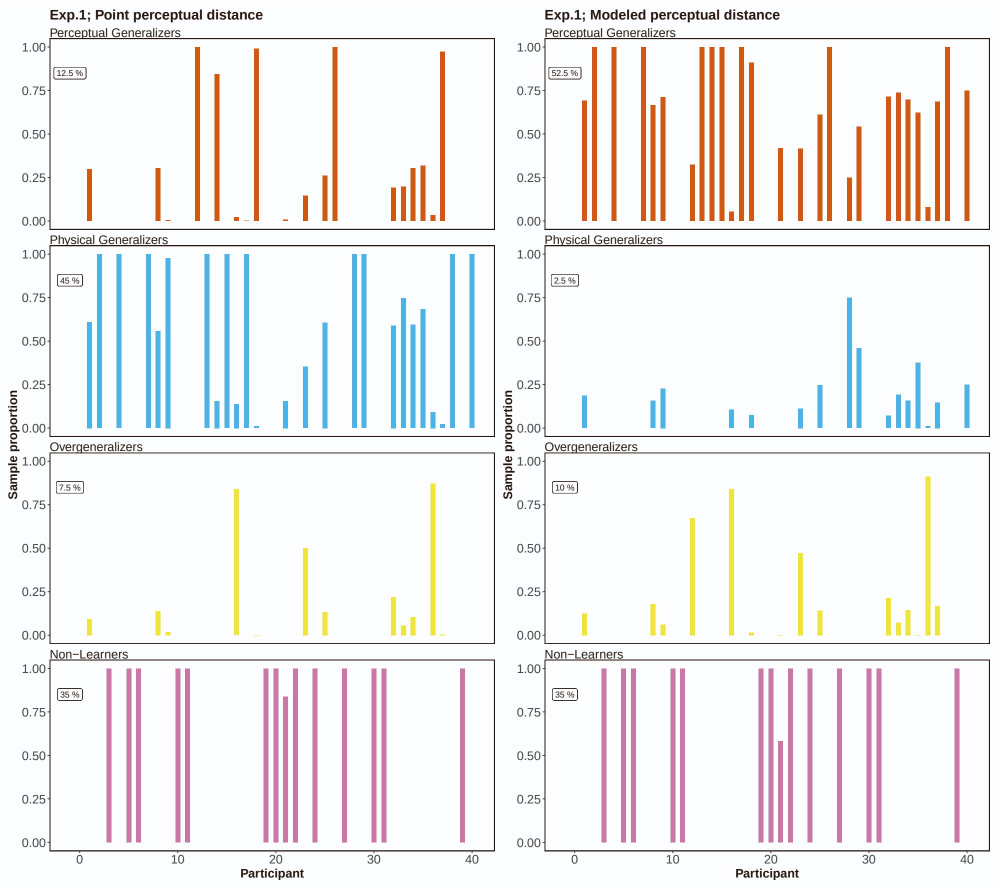

Figure S4: MCMC samples on the group allocation parameter with the new model

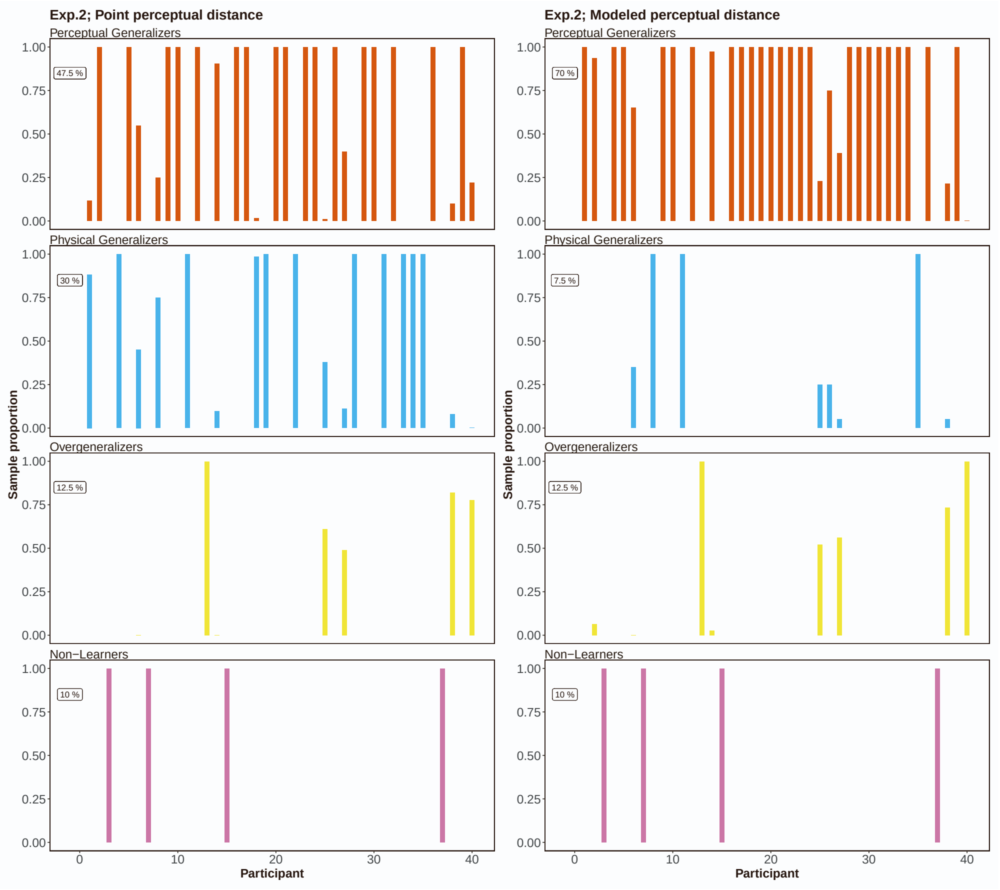

Figure S5: The Directed Acyclic Graph representing the Bayesian computational model

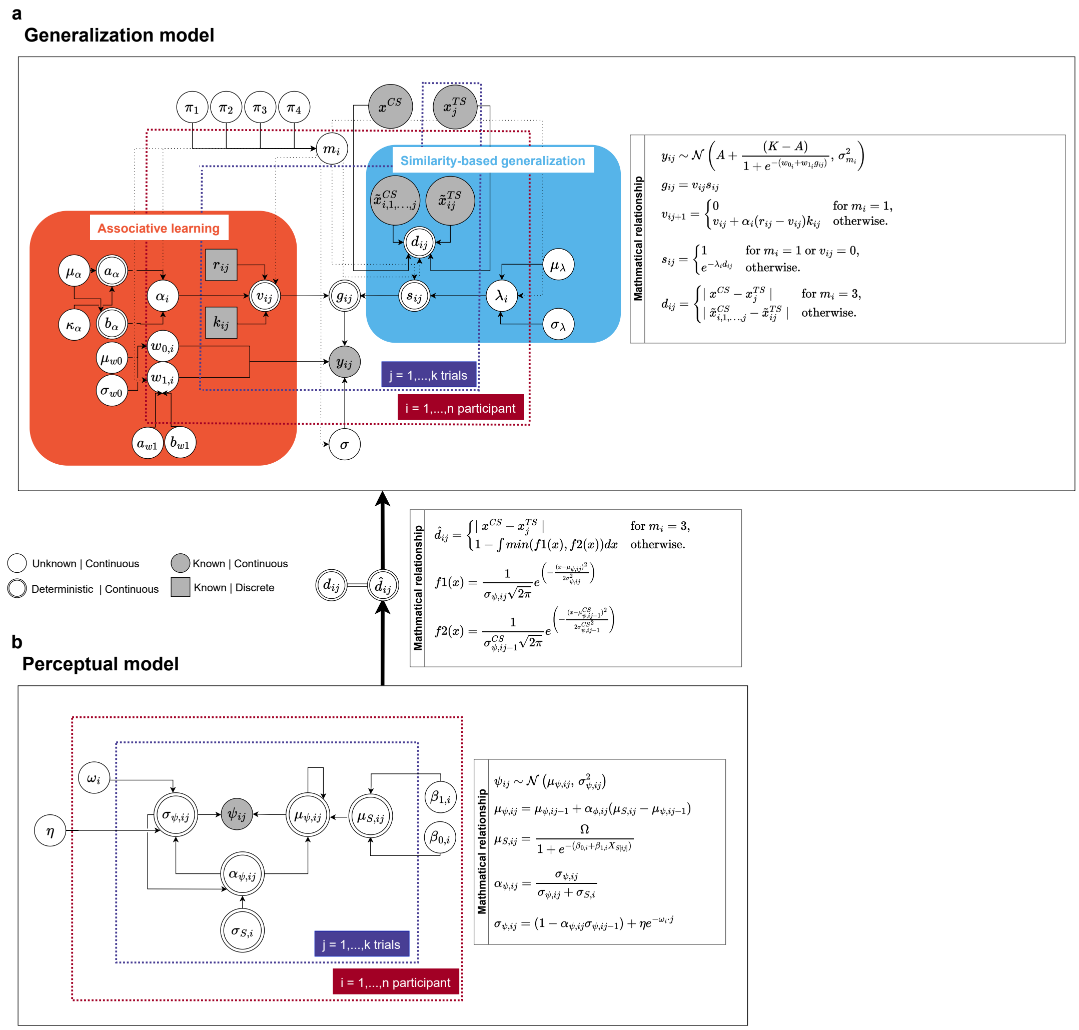

The relationship among different variables and parameters in the model is displayed. Panel a: The computational model of the generalization process. Panel b: The computational model of the perceptual process. The two processes are connected through the variable $\hat{d}_{ij}$.

Figure S6: Prior sensitivity for the perceptual model

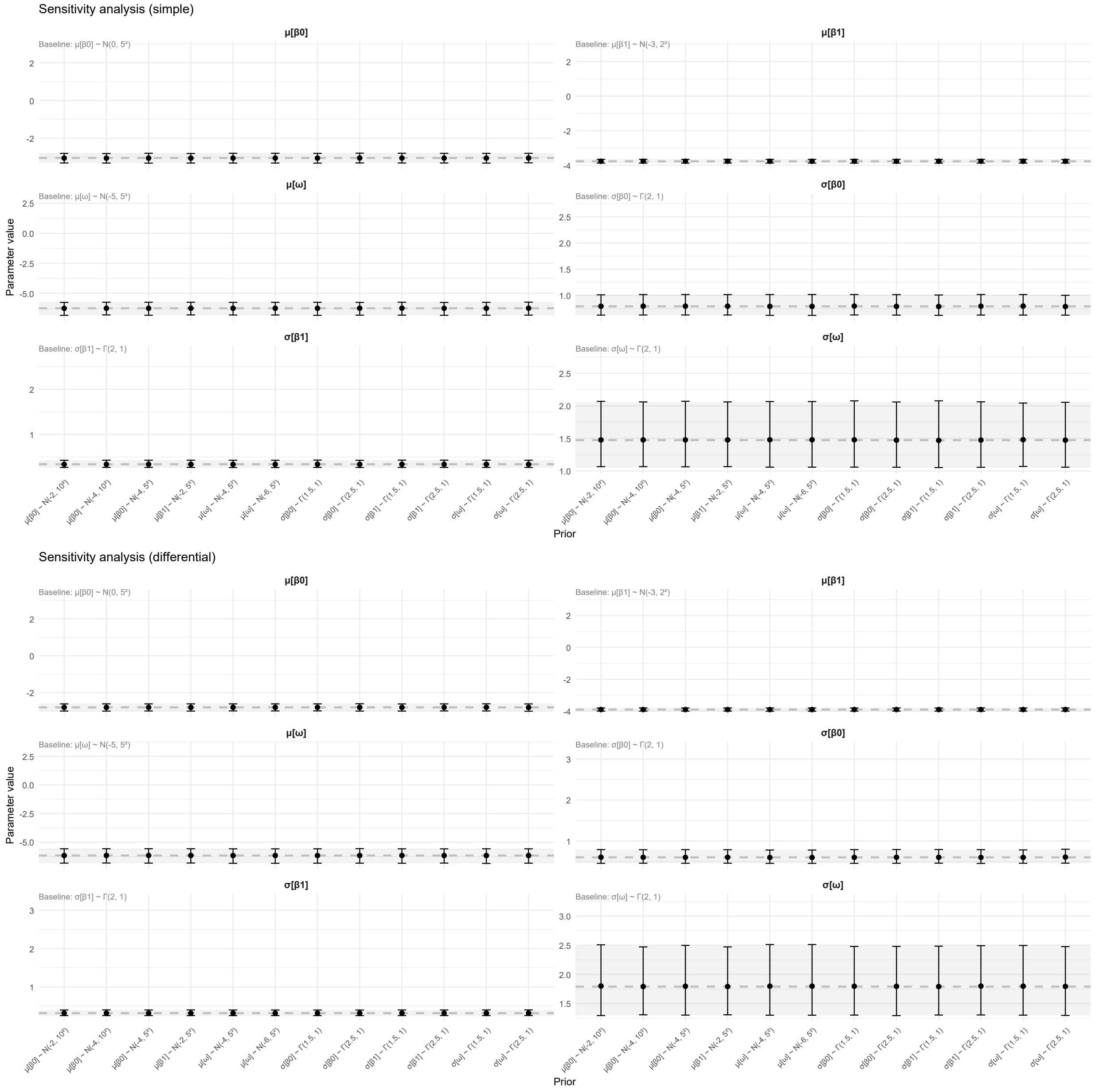

The results of prior sensitivity analysis for the perceptual model. Points represent mean parameter estimates, with error bars indicating their 95% credible intervals (2.5th to 97.5th quantiles). The grey shaded regions display the 95% credible intervals from our baseline model for comparison.

Figure S7: Prior sensitivity for the super model

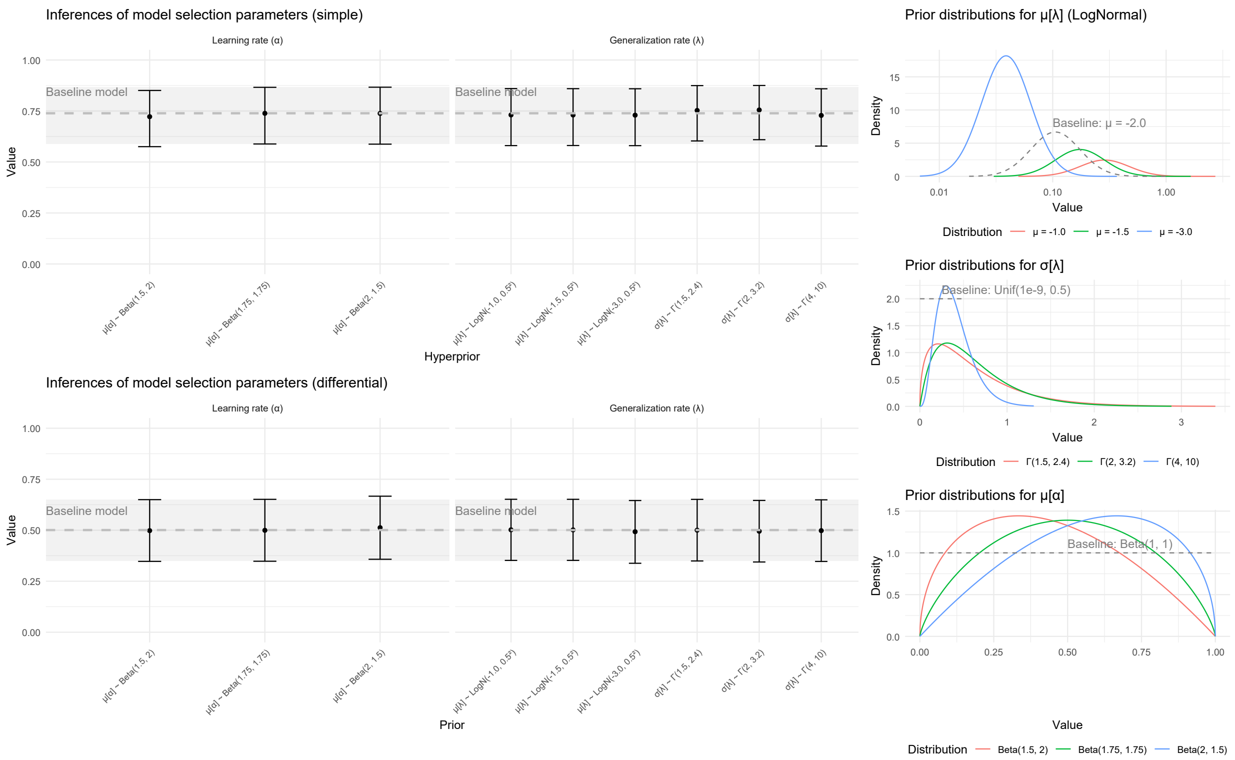

The results of prior sensitivity analysis for the super model. Points represent mean parameter estimates, with error bars indicating their 95% credible intervals (2.5th to 97.5th quantiles). The grey shaded regions display the 95% credible intervals from our baseline model for comparison.

Table S1: Priors specification for the generalization model

Parameter	Prior	Hyperprior
Learning rate	$\alpha_i = 0,\ \text{for } m_i = 1$	$\alpha_i \sim \text{Beta}(a_\alpha, b_\alpha),\ \text{otherwise}$<br>$a_\alpha = \mu_\alpha \kappa_\alpha$<br>$b_\alpha = (1-\mu_\alpha)\kappa_\alpha$<br>$\mu_\alpha \sim \text{Beta}(1,1)$<br>$\kappa_\alpha \sim \text{Uniform}(1,10)$
Generalization rate	$\lambda_i = 0,\ \text{for } m_i = 1$<br>$\lambda_i \sim N(\mu_{\lambda_{C_i}}, \sigma^2_{\lambda_{C_i}})T(10^{-9}, \lambda_{\text{limit}_{C_i}}),\ \text{for } m_i = 2$<br>$\lambda_i \sim N(\mu_\lambda, \sigma^2_\lambda)T(\lambda_{\text{limit}_{C_i}}, \infty),\ \text{otherwise}$<br>$C_1 = 1, for, m_i = 3$<br>$C_2 = 2, \text{otherwise}$	$\mu_{\lambda_1} \sim LN(-2, .5)T(0,\infty)$<br>$\mu_{\lambda_2} \sim LN(1, .5)T(0,\infty)$<br>$\sigma_{\lambda_1} \sim \text{Uniform}(10^{-9}, .5)$<br>$\sigma_{\lambda_2} \sim \text{Uniform}(10^{-9}, 3)$<br>$\lambda_{\text{limit}_1} = \frac{-ln(0.7)}{68.52}$<br>$\lambda_{\text{limit}_2} = -ln(0.7)$
Baseline response	$w_{0i} \sim N(\mu_{w_0}, \sigma^2_{w_0})$	$\mu_{w_0} \sim N(0, 10^2)$<br>$\sigma_{w_0} \sim \text{Half-Cauchy}(0,2)$
Scaling	$w_{1i} \sim \text{Gamma}(a_{w_1}, b_{w_1})$	$a_{w_1} \sim \text{Half-Cauchy}(0,2)$<br>$b_{w_1} \sim \text{Half-Cauchy}(0,2)$
Response noise		$\sigma_1 \sim \text{Uniform}(10^{-9}, 1.5),\ \text{for } m_i = 2,3,4$<br>$\sigma_2 \sim \text{Uniform}(1.5, 3),\ \text{otherwise}$
Latent group allocation	$m_i \sim \text{Multinomial}(1, \pi_1, \pi_2, \pi_3, \pi_4)$	
Group probability		$\pi_1, \pi_2, \pi_3, \pi_4 \sim \text{Dirichlet}(c(1,1,1,1))$ with $\sum_{i=1}^{4} \pi_i = 1$

Specification of priors for the generalization model. The notation $T(s,t)$ denotes truncation that limits the probability distribution to the range between s and t. The notation i denotes the participant.

Table S2: Priors specification for the perceptual model

Parameter	Prior	Hyperprior
Sensory scaling	$\beta_{0,i} \sim N(\mu_{\beta_0}, \sigma^2_{\beta_0})$<br>$\beta_{1,i} \sim LN(\mu_{\beta_1}, \sigma^2_{\beta_1})$	$\mu_{\beta_0} \sim N(0, 5^2)$<br>$\mu_{\beta_1} \sim N(-3, 2^2)$<br>$\sigma_{\beta_0} \sim \text{Half-Cauchy}(0,2)$<br>$\sigma_{\beta_1} \sim \text{Half-Cauchy}(0,2)$
Sensory uncertainty	$\sigma_{M,i} \sim LN(\mu_{\sigma_M}, \sigma^2_{\sigma_M})$	$\mu_{\sigma_M} \sim N(0, 2^2)$<br>$\sigma_{\sigma_M} \sim \text{Half-Cauchy}(0,2)$
Process noise forgetting rate	$\omega_i \sim LN(\mu_\omega, \sigma^2_\omega)$	$\mu_\omega \sim N(-5, 5^2)$<br>$\sigma_\omega \sim \text{Uniform}(10^{-9}, 1)$
Initial process noise		$\eta \sim LN(2,1)$

Specification of priors for the perceptual model.

Method S1: Parameter Recovery

Before applying the model to the empirical datasets, a simulation study was conducted. The primary objective of this simulation study was to assess the identifiability of pivotal parameters within the model through a parameter recovery study. Specifically, within the perceptual model, the focal parameters under scrutiny encompassed the sensory scaling parameters, denoted as $\beta_{0,i}$ and $\beta_{1,i}$, alongside the process noise forgetting rate ω_i and the sensory uncertainty σ_M.

To simulate the perceptual responses, we generated 180 responses for each of the 300 synthetic participants using the perceptual process model. This approach allowed us to generate a substantial dataset encompassing a total of 54,000 simulated perceptual responses, which served as the foundation for our subsequent analyses and investigations. The parameter values are simulated by the following rules: (1) the sensory scaling parameter $\beta_{0,i} \sim \text{Uniform}(-8, -1)$, (2) the sensory scaling parameter $\beta_{1,i} \sim \text{Uniform}(10^{-2}, .1)$, (3) the initial process noise $\eta = 20$, (4) the process noise forgetting rate $\omega_i \sim \text{Uniform}(10^{-4}, 10^{-1})$, (5) the sensory uncertainty $\sigma_M \sim \text{Uniform}(10^{-1}, 3)$. The simulated trials comprised a total of 180 synthetic trials, which consisted of seven distinct physical stimuli ranging from 50.8 mm to 119.42 mm, with a step size of 7.62 mm. The stimulus generation rules employed in the simulation were as follows: the first 14 trials were consistently assigned the fourth largest stimulus (96.52 mm), while the remaining trials were randomly allocated one of the seven stimuli with equal probability ($probability = \frac{1}{7}$). The stimulus order is set to identical to all 300 synthetic participants.

Method S2: Kalman Gain Analysis

In this study, we employ a one-dimensional state space model that utilizes the Kalman filter. The Kalman gain (α_ϕ) plays a crucial role in shaping the dynamic contribution of perceptual priors to the formation of current perceptions. As illustrated in Figure S2, it is evident that for the majority of participants in our experimental setup, the Kalman gain remains consistently high throughout the experiment. This suggests that sensory mapping holds a more prominent role than perceptual priors in influencing perception within the current experimental context.

Method S3: Group Allocation Patterns

In this work, we conducted an investigation to compare group allocation patterns using two distinct perceptual distance assumptions: one based on point-based perceptual distances from a previous generalization model and the other using model-based perceptual distances in the current model. Figures S3 and S4 illustrate the proportions of MCMC samples for the group allocation parameter among participants. Notably, our findings reveal a substantial increase in the allocation of Perceptual Generalizers and a substantial decrease in Physical

Generalizers for both experiments.

Method S4: Prior Specifications for Computational Models

Following a methodology similar to that of the previous study (Yu, 2023), we employed weak to non-informative priors in the current investigation. This choice reflects our limited understanding of the psychological processes under investigation. The assigned priors for both the computational model of human generalization (see Table S1) and perception (see Table S2) prioritize parameter estimates most influenced by the observed data, while exerting only weak influence on the estimates. However, we encourage future studies to empirically establish more informative priors for the underlying processes of generalization as a means of refining theoretical frameworks.

Method S5: Sensitivity Analysis of Model Priors

To assess the robustness of our findings, we conducted sensitivity analyses examining the impact of prior choices on both the perceptual model parameters and the super model that combines the two perceptual assumptions. For the perceptual model, we tested two alternative prior specifications for each parameter and compared the resulting parameter estimates with those obtained from our original priors. For the super model, we examined three different specifications of the hyperpriors governing learning and generalization rates to verify the stability of our model comparison results. These analyses help establish that our findings are not artifacts of specific prior choices but reflect genuine patterns in the data.

Method S6: Savage-Dickey Density Ratio

In the current work, we applied the Bayes factor to determine if the two generalization models that incorporate different perceptual assumptions perform differently given the experimental data. To compute the Bayes factor, we used the Savage-Dickey density ratio. Here we provide the derivation of the Savage-Dickey density ratio.

To compare two generalization models, we linearly combine the likelihood of the two models using a model selection parameter denoted as β_M. This parameter adheres to a Uniform distribution within the range [0,1]. Our primary query revolves around whether the data provides sufficient evidence to support deviation of β_M from the value 0.5, indicating that the data has distinct preferences towards the models.

Consequently, we formulate a simpler assumption H_0 for β_M to be fixed at 0.5 (designated as $\beta_{M,0}$), and a more complex assumption H_1 allowing β_M to follow a uniform distribution in the interval (0,1). The priors for other parameters, denoted as ψ, in both assumptions are

equal. This assumption guarantees the prior continuity condition: $\lim_{\beta_m \to \beta_{m_0}} P(\psi|\beta_M, H_1) = P(\psi|H_0)$.

Given the experimental data D, the marginal likelihood under H_0 is expressed as:

$$P(D|H_0) = \int P(D|\psi, H_0)P(\psi|H_0)d\psi. \tag{S1}$$

By leveraging the aforementioned continuity condition, we can alternatively express this as:

$$\begin{aligned} P(D|H_0) &= \int P(D|\psi, \beta_M = \beta_{M,0}, H_1)P(\psi|\beta_M = \beta_{M,0}, H_1)d\psi \\ &= P(D|\beta_M = \beta_{M,0}, H_1). \end{aligned} \tag{S2}$$

The Bayes rule can now be applied, leading to:

$$P(D|H_0) = \frac{P(\beta_M = \beta_{M,0}|D, H_1)P(D|H_1)}{P(\beta_M = \beta_{M,0}|H_1)}. \tag{S3}$$

Since the Bayes factor involves the ratio of marginal likelihoods, we can divide $P(D|H_0)$ as shown in equation (3) by $P(D|H_1)$, yielding:

$$\frac{P(D|H_0)}{P(D|H_1)} = \frac{P(\beta_M = \beta_{M,0}|D, H_1)}{P(\beta_M = \beta_{M,0}|H_1)}. \tag{S4}$$
